# Soft Tensor Regression

**Published:** 2021

**Authors:** Georgia Papadogeorgou, Zhengwu Zhang, David B. Dunson

**Affiliations:** Department of Statistics, University of Florida, Gainesville, FL 32611-8545, USA; Department of Statistics and Operations Research, University of North Carolina at Chapel Hill, Chapel Hill, NC 27599-3260, USA; Department of Statistical Science, Duke University, Durham, NC 27708-0251, USA

**Keywords:** adjacency matrix, Bayesian, brain connectomics, graph data, latent factors, low rank, network data, parafac, tensor regression

## Abstract

Statistical methods relating tensor predictors to scalar outcomes in a regression model generally vectorize the tensor predictor and estimate the coefficients of its entries employing some form of regularization, use summaries of the tensor covariate, or use a low dimensional approximation of the coefficient tensor. However, low rank approximations of the coefficient tensor can suffer if the true rank is not small. We propose a tensor regression framework which assumes a *soft* version of the parallel factors (PARAFAC) approximation. In contrast to classic PARAFAC where each entry of the coefficient tensor is the sum of products of row-specific contributions across the tensor modes, the soft tensor regression (Softer) framework allows the row-specific contributions to vary around an overall mean. We follow a Bayesian approach to inference, and show that softening the PARAFAC increases model flexibility, leads to improved estimation of coefficient tensors, more accurate identification of important predictor entries, and more precise predictions, even for a low approximation rank. From a theoretical perspective, we show that employing Softer leads to a weakly consistent posterior distribution of the coefficient tensor, *irrespective of the true or approximation tensor rank*, a result that is not true when employing the classic PARAFAC for tensor regression. In the context of our motivating application, we adapt Softer to symmetric and semi-symmetric tensor predictors and analyze the relationship between brain network characteristics and human traits.

## 1. Introduction

In many applications, data naturally have an array or tensor structure. When the tensor includes the same variable across two of its modes, it is often referred to as a network. Network dependence is often summarized via an adjacency matrix or tensor. For example, data might correspond to an *R* × *R* × *p* array containing *p* features measuring the strength of connections between an individual’s *R* brain regions. In tensor data analysis, interest often lies in characterizing the relationship between a tensor predictor and a scalar outcome within a regression framework. Estimation of such regression models most often requires some type of parameter regularization or dimensionality reduction since the number of entries of the tensor predictor is larger than the sample size.

In this paper, we propose a soft tensor regression (Softer) framework for estimating a high-dimensional linear regression model with a tensor predictor and scalar outcome. Softer accommodates the predictor’s tensor structure by basing the coefficient tensor estimation on the parallel factors approximation, similarly to other approaches in the literature. However, in contrast to previously developed methodology, Softer adaptively expands away from its low-rank mean to adequately capture and flexibly estimate more complex coefficient tensors. Softer’s deviations from the underlying low-rank, tensor-based structure are interpretable as variability in the tensor’s row-specific contributions.

### 1.1 Tensor Regression in the Literature

Generally, statistical approaches to tensor regression fall under the following categories: they estimate the coefficients corresponding to each tensor entry with entry-specific penalization, regress the scalar outcome on low-dimensional summaries of the tensor predictor, or estimate a coefficient tensor assuming a low-rank approximation.

A simple approach to tensor regression vectorizes the tensor predictor and fits a regression model of the outcome on the tensor’s entries while performing some form of variable selection or regularization. Examples include Cox and Savoy (2003) and Craddock et al. (2009) who employed support vector classifiers to predict categorical outcomes based on the participants’ brain activation or connectivity patterns. Other examples in neuroscience include Mitchell et al. (2004); Haynes and Rees (2005); O’Toole et al. (2005); Polyn et al. (2005) and Richiardi et al. (2011) (see Norman et al. (2006) for a review). However, this regression approach to handle tensor predictors is, at the least, unattractive, since it fails to account for the intrinsic array structure of the predictor, effectively flattening it prior to the analysis.

Alternatively, dimensionality reduction can be performed directly on the tensor predictor reducing it to low dimensional summaries. In such approaches, the expectation is that these summaries capture all essential information while decreasing the number of parameters to be estimated. For example, Zhang et al. (2019) and Zhai and Li (2019) use principal component analysis to extract information on the participants’ structural and functional brain connectivity, and use these principal components to study the relationship between brain network connections and outcomes within a classic regression framework. However, this approach could suffer due to its unsupervised nature which selects principal components without examining their relationship to the outcome. Moreover, the performance of the low-dimensional summaries is highly dependent on the number and choice of those summaries, and the interpretation of the estimated coefficients might not be straightforward.

Ginestet et al. (2017) and Durante and Dunson (2018) developed hypothesis tests for differences in the brain connectivity distribution among subgroups of individuals, and employed them to study the relationship between categorical outcomes and binary network measurements. Even though related, such approaches do not address our interest in building regression models with tensor predictors.

An attractive approach to tensor regression performs dimension reduction on the coefficient tensor. Generally, these approaches exploit a tensor’s Tucker decomposition (Tucker, 1966) and its restriction known as the parallel factors (PARAFAC) or canonical decomposition. According to the PARAFAC, a tensor is the sum of *D* rank-1 tensors, and each entry can be written as the sum of *D* products of row-specific elements. The minimum value of *D* for which that holds is referred to as the tensor’s rank. Note that the word “row” along a tensor mode is used here to represent rows in the classic matrix sense (slice of the tensor along the first mode), columns (slice of the tensor along the second mode), or slices along higher modes.

Within the frequentist paradigm, Hung and Wang (2013) suggested a bi-linear logistic regression model in the presence of a matrix predictor. For a tensor predictor, Zhou et al. (2013) and Li et al. (2018) exploited the PARAFAC and Tucker decompositions respectively, and proposed low rank approximations to the coefficient tensor. Guhaniyogi et al. (2017) proposed a related Bayesian tensor regression approach for estimating the coefficient tensor. Even though these approaches perform well for prediction in these high-dimensional tensor settings, they are bound by the approximation rank in the sense that they cannot capture any true coefficient tensor. Moreover, these approaches are not directly applicable for identifying important connections. In this direction, Wang et al. (2014) imposed sparsity in the coefficient tensor of a multi-linear logistic regression model by penalizing the PARAFAC contributions. Wang et al. (2018) proposed a related approach to identify small brain subgraphs that are predictive of an individual’s cognitive abilities. Further, Guha and Rodriguez (2021) assumed a PARAFAC decomposition of the mean of the coefficient tensor and used a spike-and-slab prior distribution to identify brain regions whose connections are predictive of an individual’s creativity index.

Low-rank approximations to the coefficient tensor of a linear tensor regression model provide a supervised approach to estimating the relationship between a tensor predictor and a scalar outcome. However, such approximations can lead to a poorly estimated coefficient tensor, misidentification of important connections, and inaccurate predictions, if the true rank of the coefficient tensor is not small. As we will illustrate in Section 3, this performance issue arises due to the inflexibility of the PARAFAC approximation which specifies that each row has a *fixed* contribution to all coefficient entries that involve it, leading to an overly rectangular or block structure of the estimated coefficient tensor. If the true coefficient tensor does not exhibit such a block structure, a large number of components *D* might be necessary in order to adequately approximate it. Due to this rigid structure, we refer to the PARAFAC approximation to estimating the coefficient tensor as the *hard* PARAFAC.

Recently, there have been efforts to relax the hard PARAFAC structure employing non-parametric methodology using kernels, Gaussian-processes, or neural networks (Signoretto et al., 2013; Suzuki et al., 2016; Kanagawa et al., 2016; Imaizumi and Hayashi, 2016; Maruhashi et al., 2018). Even though these methods are very promising, we focus on regression models which include the tensor predictor linearly, and focus on flexibly estimating the linear functional. The ideas of PARAFAC *softening* presented here could be extended to non-linear settings and situations outside the scope of tensor-regression.

### 1.2 Our Contribution

With our main focus being improved estimation and inference over model coefficients, we address the inflexibility of the hard PARAFAC in the linear tensor regression setting. Towards this goal, we propose a hierarchical modeling approach to estimate the coefficient tensor. Similarly to the hard PARAFAC, each entry of the coefficient tensor is the sum of products of row-specific contributions. However, our model specification allows *a row’s contribution* to the coefficients that involve it to be *entry-specific*. Then, the mean of the entry-specific contributions along a tensor row can be thought of as a row’s overall importance, and the entry-specific deviations as small variations in the row’s importance when interacting with the rows of the other tensor modes. Allowing for the row contributions to vary by entry leads to the softening of the hard structure in the PARAFAC approximation, and for this reason, we refer to it as the *soft* PARAFAC. We refer to the tensor regression model that uses the soft PARAFAC for estimation of the coefficient tensor as *Soft Tensor Regression* (Softer).

We follow a fully Bayesian approach to inference which allows for straightforward uncertainty quantification in the coefficient estimates and predictions. By studying the induced prior distribution on the coefficient tensor, we choose sensible values for the hyperparameters. Importantly, the flexible structure of the soft PARAFAC allows for Softer to capture *any true coefficient tensor* without increasing the base rank, in contrast to existing models that use strictly low-rank approximations. We explicitly show this by proving that the imposed prior structure on the coefficient tensor has full support on a large class including true tensors of *any rank*, and its posterior distribution is consistent for *any* approximation rank used. We also illustrate it in simulations, where we show that the performance of Softer is robust to the choice of the approximation rank. Due to its increased flexibility, Softer performs better than strictly low-rank models in identifying important entries of the tensor predictor. We extend Softer to generalized linear models and symmetric tensors, widening its applicability.

We use the soft tensor regression framework in a study of the relationship between brain structural connectomics and human traits for participants in the Human Connectome Project (HCP; Van Essen et al. (2013)). In the last few years, the HCP has played a very important role in expanding our understanding of the human brain by providing a database of anatomical and functional connections and individual demographics and traits on over a thousand healthy subjects. Data availability and increased sample sizes have allowed researchers across various fields to develop and implement new tools in order to analyze these complex and rich data (see Cole et al. (2014); McDonough and Nashiro (2014); Smith et al. (2015); Riccelli et al. (2017); Croxson et al. (2018) among many others). Using data from the HCP, exploiting state-of-the-art connectomics processing pipelines (Zhang et al., 2018), and within an adaptation of the supervised Softer framework for symmetric tensor predictors, we investigate the relationship between structural brain connection characteristics and a collection of continuous and binary human traits.

## 2. Tensor Regression

In this section, we introduce some useful notation and regression of scalar outcomes on a tensor predictor.

### 2.1 Useful Notation

Let $a\in {\mathbb{R}}^{p_{1}}$ and $b\in {\mathbb{R}}^{p_{2}}$. Then $a⊗b\in {\mathbb{R}}^{p_{1}\times p_{2}}$ is used to represent the outer product of *a* and *b* with dimension *p*_1_ × *p*_2_ and entries [*a* ⊗ *b*]_*ij*_ = *a*_*i*_*b*_*j*_. Similarly, for vectors $a_{k}\in {\mathbb{R}}^{p_{k}}$, *k* = 1, 2, …, *K*, the outer product *a*_1_ ⊗ *a*_2_ ⊗ ⋯ ⊗ *a*_*K*_ is a *K*-mode tensor ***A*** of dimensions *p*_1_, *p*_2_, …, *p*_*K*_ and entries $A_{j_{1}j_{2}\ldots j_{K}}=\prod_{k=1}^{K}a_{k,j_{k}}$. For two tensors ***A***_1_, ***A***_2_ of the same dimensions, we use ***A***_1_ ◦ ***A***_2_ to represent the Hadamard product, defined as the element-wise product of the two tensors. Further, we use 〈***A***_1_, ***A***_2_〉_*F*_ to represent the Frobenius inner product, which is the sum of the elements of ***A***_1_ ◦ ***A***_2_. When the tensors are vectors (1-mode), the Frobenius inner product is the classic dot product.

For a *K*-mode tensor ***A*** of dimensions *p*_1_, *p*_2_, …, *p*_*K*_, we use “*j*^*th*^ slice of ***A*** along mode *k*” to refer to the (*K* − 1)-mode tensor ***G*** with dimensions *p*_1_, …, *p*_*k*−1_, *p*_*k*+1_, …, *p*_*K*_ and entries $G_{j_{1}\ldots j_{k-1}j_{k+1}\ldots j_{K}}=A_{j_{1}\ldots j_{k-1}jj_{k+1}\ldots j_{K}}$. For example, the *j*^*th*^ slice of a *p*_1_ × *p*_2_ matrix along mode 1 is the matrix’s *j*^*th*^ row. As a result, we refer to “slice-specific” quantities as “row-specific” even when that slice is not along mode 1. For example, for a *p*_1_ × *p*_2_ matrix, the mean entry of the *j*^*th*^ row along mode 2 is the mean of the *j*^*th*^ column. Remembering that we use “row” to refer to slices (and not necessarily to rows in the classic matrix sense) will be useful when discussing the hard PARAFAC in Section 3 and when introducing the soft PARAFAC in Section 4.

### 2.2 Regression of Scalar Outcome on Tensor Predictor

Let *Y*_*i*_ be a continuous outcome, ***C***_*i*_ = (*C*_*i*1_, *C*_*i*2_, …, *C*_*ip*_)^*T*^ scalar covariates, and ***X***_*i*_ a *K-*mode tensor of dimensions *p*_1_, *p*_2_, …, *p*_*K*_ with entries ${\left[X_{i}\right]}_{j_{1}j_{2}\ldots j_{K}}=X_{i,j_{1}j_{2}\ldots j_{K}}$, for unit *i* = 1, 2, … *N*. Even though our model is presented here for continuous outcomes, the relationship between tensor predictors and binary or categorical outcomes can be similarly evaluated by considering an appropriate link function as we do in Section 6. We study the relationship between the outcome and the scalar and tensor predictors by assuming a linear model

(1) $$Y_{i}=\mu +C_{i}^{T}\delta +\overset{p_{1}}{\underset{j_{1}=1}{\sum }}\overset{p_{2}}{\underset{j_{2}=1}{\sum }}\cdots \overset{p_{K}}{\underset{j_{K}=1}{\sum }}X_{i,j_{1}j_{2}\ldots j_{K}}\beta_{j_{1}j_{2}\ldots j_{K}}+\epsilon_{i},\epsilon_{i}\sim N\left(0,\tau^{2}\right),$$

where $\delta \in {\mathbb{R}}^{p}$ and $\beta_{j_{1}j_{2}\ldots j_{K}}\in \mathbb{R}$. Alternatively, organizing all coefficients $\beta_{j_{1}j_{2\cdots }}j_{K}$ in a tensor ***B*** of equal dimensions to ***X*** and *j*_1_*j*_2_ … *j*_*K*_ entry equal to $\beta_{j_{1}j_{2\cdots }}j_{K}$, the same model can be written as

(2) $$Y_{i}=\mu +C_{i}^{T}\delta +{\langle X_{i},B\rangle }_{F}+\epsilon_{i}.$$

Since the coefficient tensor ***B*** includes $\prod_{k=1}^{K}p_{k}$ coefficients, it is infeasible to estimate it without some form of regularization or additional structure. Penalization or variable selection approaches based on the vectorization of the tensor predictor are implemented directly on the model of Equation 1, ignoring the predictor’s tensor structure. Alternatively, an approach to account for the predictor’s inherent structure is to assume a low-rank approximation to ***B*** based on the PARAFAC decomposition, discussed in Section 3.

## 3. Tensor Regression Using the Hard PARAFAC Approximation

Under the PARAFAC decomposition, a tensor $B\in {\mathbb{R}}^{p_{1}\times p_{2}\times \ldots p_{K}}$ can be written as

(3) $$B=\overset{D}{\underset{d=1}{\sum }}\beta_{1}^{\left(d\right)}⊗\beta_{2}^{\left(d\right)}⊗\cdots ⊗\beta_{K}^{\left(d\right)}$$

for some integer *D* and $\beta_{k}^{\left(d\right)}\in {\mathbb{R}}^{p_{k}}$. The minimum value of *D* for which ***B*** equals its representation in Equation 3 is referred to as its rank. For matrices (2−mode tensors), this decomposition is equivalent to the singular value decomposition, and *D* is the matrix rank. We refer to the *d*^*th*^ term in the sum as the PARAFAC’s *d*^*th*^ *component*.

The tensor PARAFAC decomposition leads to a natural approximation of the coefficient tensor in Equation 2 by assuming that it is in the form of Equation 3 for some *small* value of *D*, potentially much smaller than its true rank. Then, the $\prod_{k=1}^{K}p_{k}$ coefficients in ***B*** are approximated using $D\sum_{k=1}^{K}p_{k}$ parameters leading to a large reduction in the number of quantities to be estimated.

However, this reduction in the number of parameters might come at a substantial price if the rank *D* used in the approximation is smaller than the tensor’s true rank. According to Equation 3, the (*j*_1_*j*_2_ … *j*_*K*_) entry of ***B*** is equal to

(4) $$B_{j_{1}j_{2}\ldots j_{K}}=\overset{D}{\underset{d=1}{\sum }}\beta_{1,j_{1}}^{\left(d\right)}\beta_{2,j_{2}}^{\left(d\right)}\ldots \beta_{K,j_{K}}^{\left(d\right)}.$$

In turn, Equation 4 implies that row *j*_*k*_ along mode *k* has *fixed importance*, expressed as fixed row contributions $\beta_{k,j_{k}}^{\left(d\right)}$, to all coefficient entries $B_{j_{1}j_{2}\ldots j_{K}}$ that include *j*_*k*_, irrespective of the remaining indices. We refer to $\beta_{k,j_{k}}^{\left(d\right)}$ as the *d*^*th*^ *j*_*k*_-row contribution along mode *k*. This is best illustrated by considering a rank-1 2-mode tensor (matrix) ***B*** = *β*_1_ ⊗ *β*_2_ for vectors $\beta_{1}\in {\mathbb{R}}^{p_{1}}$ and $\beta_{2}\in {\mathbb{R}}^{p_{2}}$. Then, $B_{j_{1}j_{2}}=\beta_{1,j_{1}}\beta_{2,j_{2}}$, and the same entry $\beta_{1,j_{1}}$ is used in $B_{j_{1}j_{2}}$ irrespective of *j*_2_. This gives rise to a *rectangular structure* in ***B*** in which a row’s importance, $\beta_{1,j_{1}}$, is fixed across all columns (and similarly for $\beta_{2,j_{2}}$).

**Figure 1:**
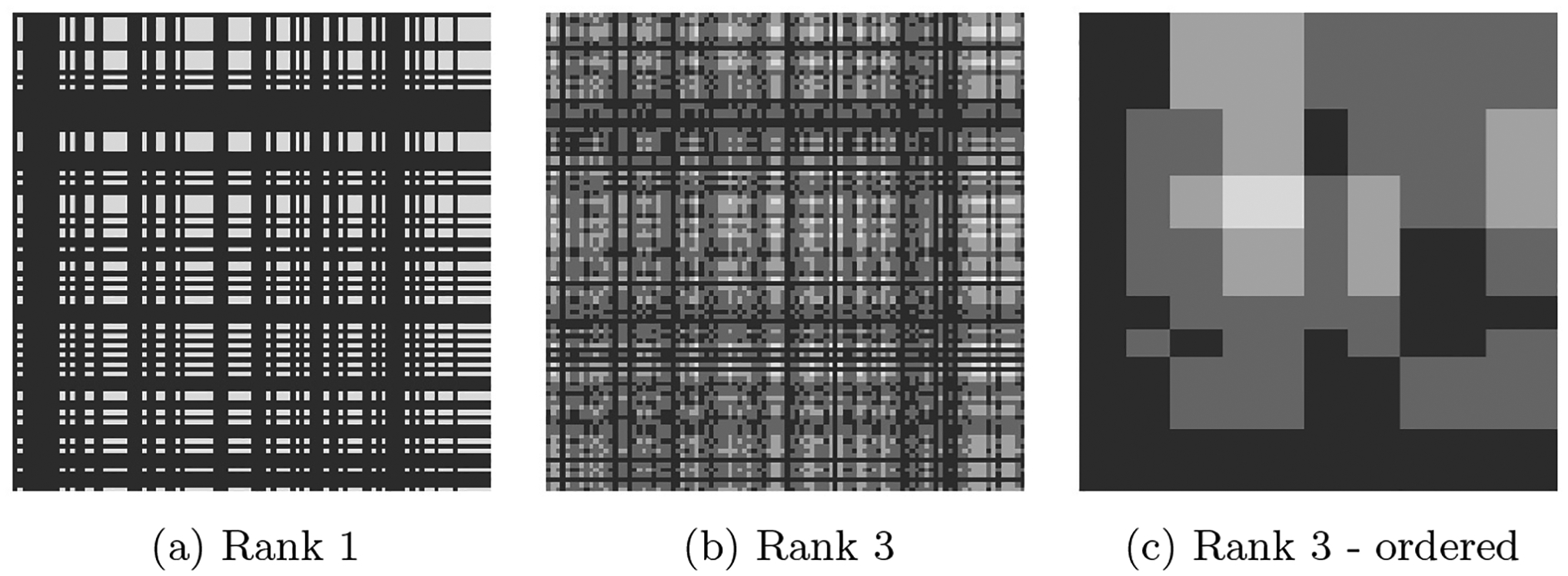
Illustration of the Hard PARAFAC Inflexibility. Panel (a) shows a rank-1 tensor of the form ***B*** = *β*_1_ ⊗ *β*_2_, for vectors *β*_1_, *β*_2_ ∈ {0, 1}^100^. Panel (b) shows a rank-3 matrix that is the sum of the three rank-1 tensors like the one in panel (a). Panel (c) shows the same rank-3 matrix with rows and columns reordered according to their mean entry.

We further illustrate this in Figure 1a where we plot *β*_1_ ⊗ *β*_2_ for randomly generated vectors *β*_1_, *β*_2_ ∈ {0, 1}^100^. It is evident from Figure 1a that rank-1 matrices are organized in a rectangular structure where rows and columns are either uniformly excluded or not. Even though the generated vectors are binary for ease of illustration, the rectangular structure persists even when *β*_1_, *β*_2_ include non-binary entries. The rectangular structure observed in rank-1 tensors indicates that a rank-1 (*D* = 1) approximation to the coefficient tensor could be quite limiting. Generally, a rank-*D* approximation for *D* > 1 is employed to estimate the coefficient tensor. Figure 1b shows a matrix ***B*** of rank *D* = 3, summing over three rank-1 tensors like the one in Figure 1a. The rank-3 tensor alleviates but does not annihilate the rectangular structure observed previously. This is most obvious in Figure 1c where the rows and columns of Figure 1b are re-ordered according to their mean entry. In Appendix B we further demonstrate the inflexibility of the hard PARAFAC’s block structure.

The said block structure is also evident in the work by Zhou et al. (2013); Guhaniyogi et al. (2017) and Li et al. (2018) where they simulated data based on binary coefficient matrices. When these matrices represent combinations of rectangles (such as squares or crosses), the approximation performed well in estimating the true coefficient tensor. However, in situations where the true coefficient tensor was irregular, an increase in the rank was necessary in order to vaguely approximate the truth.

## 4. Soft Tensor Regression

Our approach proceeds by increasing the number of parameters in the regression model in Equation 2 and subsequently imposing sufficient structure to ensure model regularization and adaptability, simultaneously. It borrows from the low-rank structure of the hard PARAFAC, but it is more flexible than the rigid form illustrated in Equation 4 and Figure 1, and can adaptively expand away from low-rank. We introduce tensors $B_{k}^{\left(d\right)}$ of equal dimensions to ***B*** and write

(5) $$B=\overset{D}{\underset{d=1}{\sum }}B_{1}^{\left(d\right)}\circ B_{2}^{\left(d\right)}\circ \ldots \circ B_{K}^{\left(d\right)}.$$

From Equation 5, the coefficient with indices $\underset{˜}{j}=\left(j_{1}j_{2}\ldots j_{K}\right)$ is written as the sum of *D* products of *K* parameters

$$B_{\underset{˜}{j}}=\overset{D}{\underset{d=1}{\sum }}\beta_{1,\underset{˜}{j}}^{\left(d\right)}\beta_{2,\underset{˜}{j}}^{\left(d\right)}\ldots \beta_{K,\underset{˜}{j}}^{\left(d\right)},$$

where $\beta_{k,\underset{˜}{j}}^{\left(d\right)}$ is the ${\underset{˜}{j}}^{th}$ entry of the tensor $B_{k}^{\left(d\right)}$. For reasons that will become apparent later, the parameters $\beta_{k,\underset{˜}{j}}^{\left(d\right)}$ are referred to as the $j_{k}^{th}$ row-specific contributions along mode *k*. Note that these row-specific contributions are allowed to depend on all indices $\underset{˜}{j}$. For unrestricted $B_{k}^{\left(d\right)}\text{s}$, Equation 5 does not impose any restrictions and any tensor ***B*** can be written in this form (take *D* = 1, $B_{1}^{\left(1\right)}=B$ and $B_{k}^{\left(1\right)}=1$, for all *k* > 1).

Writing the coefficient tensor as in Equation 5 might appear counter-productive at first since the already high-dimensional problem of estimating the $\prod_{k=1}^{K}p_{k}$ parameters in ***B*** is translated to an *even higher*-dimensional problem with $DK\prod_{k=1}^{K}p_{k}$ parameters in the right-hand side of Equation 5. However, as we will see, that is not a problem if adequate structure is imposed on the tensors $B_{k}^{\left(d\right)}$. In fact, in Section 4.1 we show that the hard PARAFAC can be written in this form for carefully designed tensors $B_{k}^{\left(d\right)}$, which shows that sufficient structure on the tensors $B_{k}^{\left(d\right)}$ can lead to drastic dimensionality reduction. In Section 4.2, we present our approach which is based on imposing structure on the tensors $B_{k}^{\left(d\right)}$ in a careful manner such that it simultaneously allows for low-dimensional and flexible estimation of ***B***. We refer to the resulting characterization as the soft PARAFAC of ***B***.

### 4.1 Representation of the Hard PARAFAC Motivating the Soft PARAFAC

As shown in Equation 4, the hard PARAFAC row-specific contributions to each entry of the coefficient tensor are fixed across the remaining indices. Hence, the hard PARAFAC can be written in the form Equation 5 by specifying tensors $B_{k}^{\left(d\right)}$ of which two entries with the same *j*_*k*_ index are equal:

$${\left[B_{k}^{\left(d\right)}\right]}_{j_{1}j_{2}\ldots j_{k}\ldots j_{K}}={\left[B_{k}^{\left(d\right)}\right]}_{j_{1}^{\prime }\ldots j_{k-1}^{\prime }j_{k}j_{k+1}^{\prime }\ldots j_{K}^{\prime }}.$$

**Figure 2:**
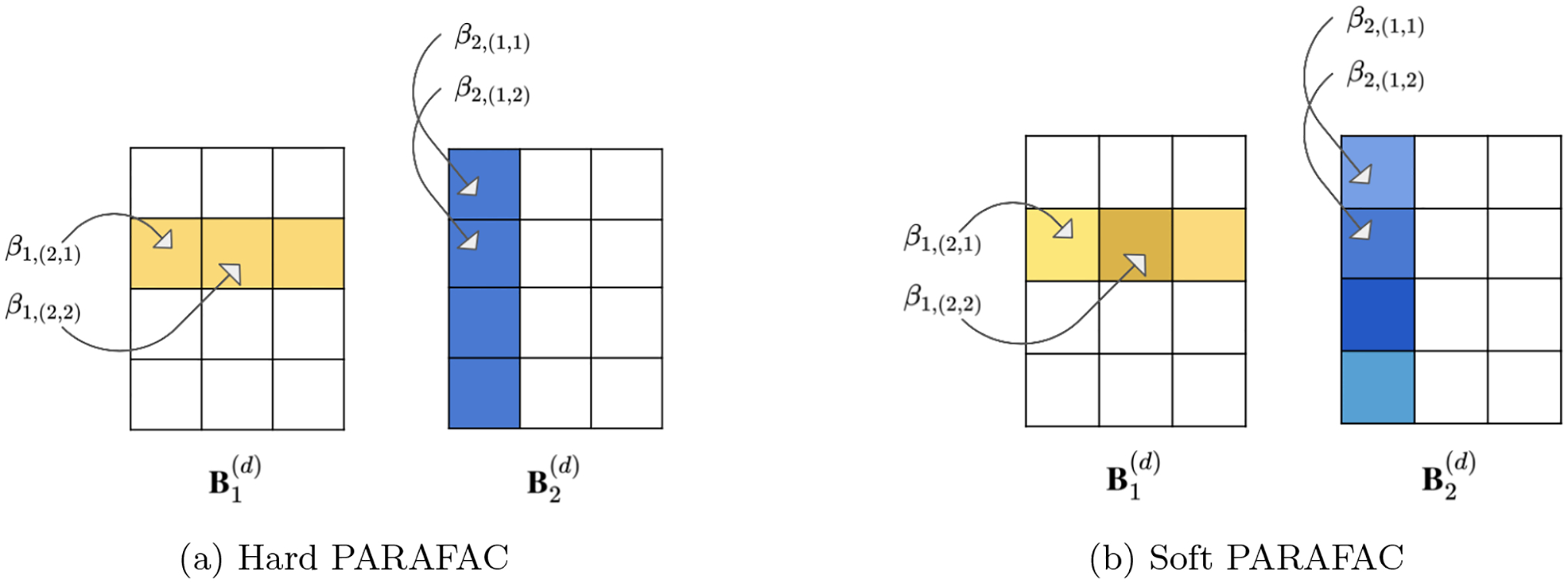
Row-specific Contributions for the Hard and Soft PARAFAC. Left: For the hard PARAFAC, the contributions are fixed across remaining indices. Right: For the soft PARAFAC, the contributions of a row and column are entry specific and centered around an overall mean.

This structure on the tensors $B_{k}^{\left(d\right)}$ can be visualized as *p*_*k*_ *constant* slices along mode *k* representing the fixed row-specific contributions to all coefficient entries that involve it. This structure is illustrated in Figure 2a for a 4-by-3 coefficient matrix. As an example, the contribution of row 2 along mode 1 is constant (*β*_1,(2,1)_ = *β*_1,(2,2)_ = *β*_1,(2,3)_), and the same is true for the contribution of row 1 along mode 2 (*β*_2,(1,1)_ = *β*_2,(2,1)_ = *β*_2,(3,1)_ = *β*_2,(4,1)_).

This demonstrates that the hard PARAFAC is one example of structure that can be imposed on the $B_{k}^{\left(d\right)}\text{s}$ in order to approximate ***B***. The connection between Equation 5 and the hard PARAFAC is the reason why we refer to $\beta_{k,\underset{˜}{j}}^{\left(d\right)}$ as row-specific contributions along mode *k*. However, the hard PARAFAC structure is quite strict and it imposes equalities across the *p*_*k*_ slices of $B_{k}^{\left(d\right)}$. Since the hard PARAFAC can only capture coefficient tensors of rank up to *D*, the hard PARAFAC’s rigid structure on the tensors $B_{k}^{\left(d\right)}$ can severely limit the flexibility of the model to capture a true coefficient tensor ***B*** of higher rank.

### 4.2 The Soft PARAFAC

The soft PARAFAC builds upon the hard PARAFAC’s low-rank structure, while providing additional flexibility by introducing entry-specific variability in the row contributions. Instead of forcing all entries of $B_{k}^{\left(d\right)}$ with the same index *j*_*k*_ to be equal to each other, the soft PARAFAC assumes that the entries of $B_{k}^{\left(d\right)}$ are centered around a *j*_*k*_-specific value, $\gamma_{k,j_{k}}^{\left(d\right)}$. Specifically, the soft PARAFAC specifies that, for all *k* = 1, 2, …, *K*, *j*_*k*_ = 1, 2, …, *p*_*k*_, and *d* = 1, 2, … *D*,

(6) $$\beta_{k,\underset{˜}{j}}^{\left(d\right)}\sim N\left(\gamma_{k,j_{k}}^{\left(d\right)},\sigma_{k}^{2}\zeta^{\left(d\right)}\right),$$

for some $\gamma_{k,j_{k}}^{\left(d\right)}\in \mathbb{R}$, $\sigma_{k}^{2}$, *ζ*^(*d*)^ > 0, where $\underset{˜}{j}$ is the (*j*_1_*j*_2_ … *j*_*k*_ … *j*_*K*_) entry of the tensor. The connection between the soft and hard PARAFAC is evident by noticing that the mean values *γ* depend only on the index *j*_*k*_, and not on the remaining indices in $\underset{˜}{j}$. In fact, the *γ* parameters resemble the *j*_*k*_-specific entries in the hard PARAFAC, and they specify that Softer is based on an underlying *γ*-based rank-D PARAFAC:

$$E\left[B_{\underset{˜}{j}}|\Gamma ,S,Z\right]=\overset{D}{\underset{d=1}{\sum }}\gamma_{1,j_{1}}^{\left(d\right)}\gamma_{2,j_{2}}^{\left(d\right)}\ldots \gamma_{K,j_{K}}^{\left(d\right)},$$

where Γ, *S*, *Z* are the collections of the *γ*, *σ*, *ζ* parameters respectively. At the same time, Equation 6 allows variation within the mode−*k* slices of $B_{k}^{\left(d\right)}$ by considering them as random effects centered around an overall mean. This implies that row *j*_*k*_’s importance is allowed to be *entry-specific* leading to a softening in the hard PARAFAC structure. The soft PARAFAC is illustrated in Figure 2b. Here, the row-contributions are centered around a common value (a value resembling the row-contribution according to the hard PARAFAC) but are entry-specific. For example, *β*_1,(2,1)_ is similar but not equal to *β*_1,(2,2)_, *β*_1,(2,3)_.

**Figure 3:**
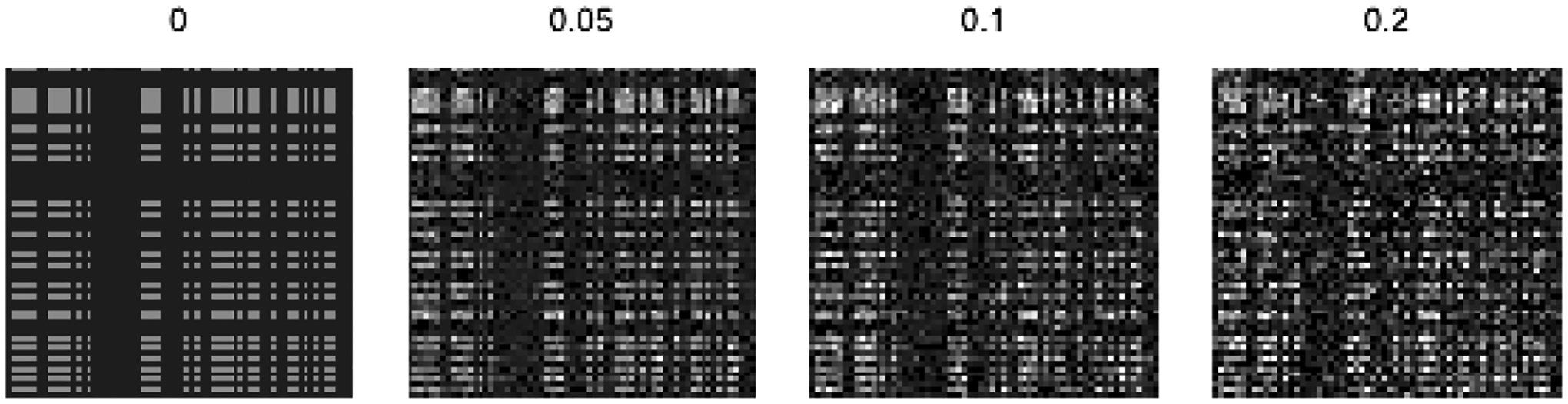
Soft PARAFAC Matrices. The plot on the left shows a rank-1 binary matrix *γ*_1_ ⊗ *γ*_2_ for *γ*_1_, *γ*_2_ ∈ {0, 1}^64^. The remaining matrices are generated according to Equation 6 for variance equal to 0.05, 0.1, and 0.2.

The entry-specific contributions deviate from the baseline according to a mode-specific parameter, $\sigma_{k}^{2}$, and a component-specific parameter, *ζ*^(*d*)^. As we will discuss later, the inclusion of *ζ*^(*d*)^ in the variance forces a larger amount of shrinkage on the entry-specific importance for components *d* that have limited overall importance. For $\sigma_{k}^{2}\zeta^{\left(d\right)}=0$ the soft PARAFAC reverts back to the hard PARAFAC, with row-specific contributions fixed at $\gamma_{k,j_{k}}^{\left(d\right)}$. However, larger values of $\sigma_{k}^{2}\zeta^{\left(d\right)}$ allow for a PARAFAC-based approximation that deviates from its hard underlying structure and can be used to represent *any* true tensor ***B***. This is further illustrated in Figure 3 where *γ*_1_, *γ*_2_ ∈ {0, 1}^64^, and entry-specific contributions are generated according to Equation 6 with $\sigma_{k}^{2}\zeta^{\left(d\right)}\in \left\{0,0.05,0.1,0.2\right\}$. The soft PARAFAC resembles a structured matrix though higher values of the conditional variance lead to further deviations from a low-rank structure.

The structure imposed by the soft PARAFAC has an interesting interpretation. The parameters $\gamma_{k,j_{k}}^{\left(d\right)}$ represent the baseline importance of row *j*_*k*_ along the tensor’s *k*^*th*^ mode. In contrast to the hard PARAFAC, the soft PARAFAC allows for structured, tensor-based deviations of a row’s importance, by allowing row *j*_*k*_’s contribution to manifest differently based on the rows of the other modes that participate with it in a coefficient entry, $\underset{˜}{j}\setminus \left\{j_{k}\right\}$, through $\beta_{k,\underset{˜}{j}}^{\left(d\right)}$. This interpretation of the soft PARAFAC structure is coherent in network settings like the one in our brain connectomics study, where we expect a brain region to have some baseline value for its connections, but the magnitude of this importance might slightly vary depending on the other region with which these connections are made. In this sense, defining deviations from the hard PARAFAC through deviations in the row-specific contributions as specified in Equation 6 represents a *tensor-based* relaxation of the hard PARAFAC structure, which is itself tensor-based.

### 4.3 Bayesian Inference in the Soft Tensor Regression Framework

Softer is placed within the Bayesian paradigm, which allows for straightforward uncertainty quantification. We consider the structure on $B_{k}^{\left(d\right)}$ expressed in Equation 6 as part of the prior specification on the model parameters of Equation 2. Since $\gamma_{k,j_{k}}^{\left(d\right)}$ are the key building blocks for the mean of ***B*** representing the underlying hard PARAFAC, we borrow from Guhaniyogi et al. (2017) and specify

$$\gamma_{k,j_{k}}^{\left(d\right)}\sim N\left(0,\tau_{\gamma }\zeta^{\left(d\right)}w_{k,j_{k}}^{\left(d\right)}\right)$$

$$\tau_{\gamma }\sim \Gamma \left(a_{\tau },b_{\tau }\right)$$

$$w_{k,j_{k}}^{\left(d\right)}\sim Exp\left({\left(\lambda_{k}^{\left(d\right)}\right)}^{2}/2\right),$$

$$\lambda_{k}^{\left(d\right)}\sim \Gamma \left(a_{\lambda },b_{\lambda }\right)$$

ζ~Dirichlet(α/D,α/D,…,α/D),

where ***ζ*** = (*ζ*^(1)^, *ζ*^(2)^, …, *ζ*^(*D*)^). Therefore, the parameters $\gamma_{k,j_{k}}^{\left(d\right)}$ vary around 0 with variance that depends on an overall parameter *τ*_*γ*_, and component and row-specific parameters *ζ*^(*d*)^ and $w_{k,j_{k}}^{\left(d\right)}$. As discussed in Guhaniyogi et al. (2017), the row-specific components $w_{k,j_{k}}^{\left(d\right)}$ lead to an adaptive Lasso type penalty on $\gamma_{k,j_{k}}^{\left(d\right)}$ (Armagan et al., 2013), and $\gamma_{k,j_{k}}^{\left(d\right)}|\tau_{\gamma }$, *ζ*^(*d*)^, $\lambda_{k}^{\left(d\right)}$ follows a double exponential (Laplace) distribution centered at 0 with scale $\tau_{\gamma }\zeta^{\left(d\right)}/\lambda_{k}^{\left(d\right)}$ (Park and Casella, 2008).

The component-specific variance parameter *ζ*^(*d*)^ in the prior of $\gamma_{k,j_{k}}^{\left(d\right)}$ encourages only a subset of the *D* components to contribute substantially in the tensor’s PARAFAC approximation, since parameters $\gamma_{k,j_{k}}^{\left(d\right)}$ for *d* with small *ζ*^(*d*)^ are shrunk towards zero. We include *ζ*^(*d*)^ in the conditional variance of $\beta_{k,\underset{˜}{j}}^{\left(d\right)}$ in Equation 6 to ensure that shrinkage of the baseline row contributions $\gamma_{k,j_{k}}^{\left(d\right)}$ is accompanied by a shrinkage of the corresponding entry-specific contributions $\beta_{k,\underset{˜}{j}}^{\left(d\right)}$ towards zero, and that a reduction in the variance of $\gamma_{k,j_{k}}^{\left(d\right)}$ is not overcompensated by an increase in the conditional variance of $\beta_{k,\underset{˜}{j}}^{\left(d\right)}$.

We assume normal prior distributions on the intercept and scalar covariates’ coefficients (*μ*, ***δ***) ~ *N*(0, Σ_0_), and inverse gamma priors on the residual variance *τ*^2^ ~ *IG*(*a*_*τ*_, *b*_*τ*_) and the mode-specific variance components $\sigma_{k}^{2}\sim \Gamma \left(a_{\sigma },b_{\sigma }\right)$. Specific choices for the hyperparameter values are discussed in Section 4.4.

### 4.4 Choosing Hyperparameters to Achieve Desirable Characteristics of the Induced Prior

The prior distribution on the coefficient tensor ***B*** ~ *π*_***B***_ is induced by our prior specification on the remaining parameters. The choice of hyperparameters can have a large effect on model performance, and the use of diffuse, non-informative priors can perform poorly in some situations (Gelman et al., 2008). For that reason and in order to assist default choices of hyperparameters leading to weakly informative prior distributions with interpretable flexibility, we study the properties of the induced prior on ***B***.

We do so in the following way. First, in Proposition 1 we provide expressions for the induced prior expectation, variance and covariance for the entries in ***B***. These expressions illustrate the importance of certain hyperparameters in how the soft PARAFAC transitions away from its underlying, low-rank, hard version. Then, in Proposition 2 we provide default values for hyperparameters for a standardized 2-mode tensor predictor such that, a priori, $\text{Var}\left(B_{\underset{˜}{j}}\right)=V^{*}$, and the proportion of the variance that arises due to the proposed PARAFAC softening is equal to AV*. Finally, in Section 4.5 we study statistical properties of the induced prior on ***B***, and we show full support over a large class of coefficient tensors irrespective of the base rank *D* used, which leads to consistent posterior distributions. All proofs are in Appendix A.

**Proposition 1** *For* $\underset{˜}{j}$, ${\underset{˜}{j}}^{\prime }\in {⊗}_{k=1}^{K}\left\{1,2,\ldots ,p_{k}\right\}$, *such that $\underset{˜}{j}\neq {\underset{˜}{j}}^{\prime }$*, *we have that* $E\left(B_{\underset{˜}{j}}\right)=0$, $\text{Cov}\left(B_{\underset{˜}{j}},B_{{\underset{˜}{j}}^{\prime }}\right)=0$, *and for a*_*λ*_ > 2,

$$\text{Var}\left(B_{j}\right)=\left\{D\overset{K-1}{\underset{r=0}{\prod }}\frac{\alpha /D+r}{\alpha +r}\right\}\left[\overset{K}{\underset{l=0}{\sum }}\rho_{l}\left(\begin{array}{c} K\\ l \end{array}\right){\left\{\frac{2b_{\lambda }^{2}}{b_{\tau }\left(a_{\lambda }-1\right)\left(a_{\lambda }-2\right)}\right\}}^{l}{\left(\frac{a_{\sigma }}{b_{\sigma }}\right)}^{K-l}\right],$$

*where ρ*_0_ = 1 *and ρ*_*l*_ = *a*_*τ*_(*a*_*τ*_ + 1) … (*a*_*τ*_ + *l* − 1) *for l* ≥ 1.

**Remark 1** *The hyperparameters of the softening variance*, *a*_*σ*_, *b*_*σ*_*. Remember that $\sigma_{k}^{2}$ is the parameter driving the PARAFAC softening by allowing row-specific contributions to vary. From Proposition 1*, *it is evident that the prior of $\sigma_{k}^{2}$ is only influential on the first two moments of $B_{\underset{˜}{j}}$ through its mean*, $\frac{a_{\sigma }}{b_{\sigma }}$, *with higher prior expectation of $\sigma_{k}^{2}$ leading to higher prior variance of $B_{\underset{˜}{j}}$. Therefore*, *prior elicitation for a*_*σ*_, *b*_*σ*_ *could be decided based on the ratio $\frac{a_{\sigma }}{b_{\sigma }}$*.

**Remark 2** *Variance of coefficient entries for the hard PARAFAC. For $\sigma_{k}^{2}=0$*, *the prior variance of the coefficient tensor entries is equal to the prior variance of the hard PARAFAC*,

$${\text{Var}}^{\text{hard}}\left(B_{\underset{˜}{j}}\right)=\left\{D\overset{K-1}{\underset{r=0}{\prod }}\frac{\alpha /D+r}{\alpha +r}\right\}\frac{\rho_{K}}{b_{\tau }^{K}}{\left\{\frac{2b_{\lambda }^{2}}{\left(a_{\lambda }-1\right)\left(a_{\lambda }-2\right)}\right\}}^{K}.$$

Comparing the variance of $B_{\underset{˜}{j}}$ based on the soft and hard PARAFAC quantifies the amount of additional flexibility that is provided by the PARAFAC softening, expressed as

$$AV=\frac{\text{Var}\left(B_{\underset{˜}{j}}\right)-{\text{Var}}^{\text{hard}}\left(B_{\underset{˜}{j}}\right)}{\text{Var}\left(B_{\underset{˜}{j}}\right)}\in [0,1).$$

We refer to this quantity as the *additional variance*. Motivated by Figure 3, hyperparameters should be chosen such that the induced prior assigns more weight to coefficient tensors that resemble low-rank factorizations, and achieves sufficiently but not overly large variance on the regression coefficients. Proposition 2 provides such conditions on the hyperparameters for matrix predictors (*K* = 2). For a tensor predictor with *K* > 2, similar conditions on the hyperparameters can be acquired by following similar steps.

**Proposition 2** *For a matrix predictor*, target variance V* ∈ (0, ∞), target additional variance AV* ∈ [0, 1), *if the hyperparameters satisfy a*_*λ*_ > 2,

(7) $$\frac{2b_{\lambda }^{2}}{\left(a_{\lambda }-1\right)\left(a_{\lambda }-2\right)}=\frac{b_{\tau }}{a_{\tau }}\sqrt{\frac{V^{*}\left(1-AV^{*}\right)a_{\tau }}{C\left(a_{\tau }+1\right)}}$$

and

(8) $$\frac{a_{\sigma }}{b_{\sigma }}=\sqrt{\frac{V^{*}\left(1-AV^{*}\right)a_{\tau }}{C\left(a_{\tau }+1\right)}}\left\{\sqrt{1-\frac{a_{\tau }+1}{a_{\tau }}\left\{1-{\left(1-AV^{*}\right)}^{-1}\right\}}-1\right\},$$

*where C* = (*α*/*D* + 1)/(*α* + 1), then we have that a priori $\text{Var}\left(B_{\underset{˜}{j}}\right)=V^{*}$, *and AV* = AV*.

Proposition 2 is used in our simulations and study of brain connectomics to choose hyperparameters such that, a priori, $\text{Var}\left(B_{\underset{˜}{j}}\right)=1$ and *AV* = 10%, assuming a tensor predictor with standardized entries. Specifically, we set *a*_*τ*_ = 3, *a*_*σ*_ = 0.5 and calculate the values of *b*_*τ*_, *b*_*σ*_ for which V* = 1 and AV* = 10%. These values correspond to $b_{\tau }\approx 6.3\sqrt{C}$ and $b_{\sigma }\approx 8.5\sqrt{C}$. We specify *α*_*σ*_ = 0.5 < 1 to encourage, a priori, smaller values of $\sigma_{k}^{2}$. Through-out, we use *α* = 1 and *D* = 3, unless stated otherwise. For the hyperparameters controlling the underlying hard PARAFAC, we specify *a*_*λ*_ = 3 and $b_{\lambda }=\sqrt[{2K}]{a_{\lambda }}$. Lastly, assuming centered and scaled outcome and scalar covariates, we specify ${\left(\mu ,\delta^{T}\right)}^{T}\sim N\left(0,I_{p+1}\right)$, and residual variance *τ*^2^ ~ *IG*(2, 0.35) which specifies *P*(*τ*^2^ < 1) ≈ 0.99.

**Remark 3** *Interplay between variance hyperparameters. From* Equation 8, *we see that the prior mean of $\sigma_{k}^{2}$ depends on the target variance and the proportion of that variance that is attributable to the PARAFAC softening*, *and does not depend on the remaining hyperparameters (considering that a*_*τ*_/(*a*_*τ*_ + 1) ≈ 1*). Furthermore*, Equation 7 *only includes hyperparameters which drive the underlying hard PARAFAC*, and it depends on V* and AV* only through V*(1 − AV*), *which expresses the prior variability in* ***B*** *that is not attributed to the PARAFAC softening. Therefore*, *in Softer there is a clear and desirable separation between the hard and soft PARAFAC variance hyperparameters. Lastly*, *since $\frac{2b_{\lambda }^{2}}{\left(a_{\lambda }-1\right)\left(a_{\lambda }-2\right)}$ is the prior mean of $w_{k,j_{k}}^{\left(d\right)}$*, Equation 7 illustrates the interplay between the two components in the variance V*(1 − AV*) *of the underlying hard PARAFAC structure: when the prior mean of τ*_*γ*_ *increases*, the prior mean of $w_{k,j_{k}}$ has to decrease in order to maintain the target variance due to the underlying PARAFAC at V*(1 − AV*).

### 4.5 Posterior Consistency and Softer’s Robustness to the Underlying Rank

In this section, we focus on the choice of the rank *D* for Softer. We discuss that results based on Softer are likely robust to small changes in the choice of the underlying rank. To support this claim, we first provide two intuition-based arguments, and then a theoretical one. Finally, Softer’s robustness to the choice of *D* is empirically illustrated in simulated examples in Section 5.

When employing the hard PARAFAC, Guhaniyogi et al. (2017) recommended using *D* = 10 for a predictor of dimension 64 × 64. The reason is that the prior on ***ζ*** permits some amount of flexibility in the number of components that contribute to the coefficient matrix approximation in that (a) if the matrix can be well-approximated by a rank lower than *D*, the prior leads to a reduction in the approximation’s effective rank, and (b) if all *D* components are useful in estimation, all of them acquire sufficient weight. Since Softer employs the same prior on ***ζ***, it also allows for sparsity in the effective components in the underlying hard PARAFAC, in that if the true coefficient tensor is of rank lower than *D*, higher order components will be heavily shrunk towards zero, and softening will be minimal. This claim will be further supported in simulations in Section 5.1 where we illustrate that Softer reverts back to the hard PARAFAC when the underlying low-rank structure is true.

**Figure 4:**
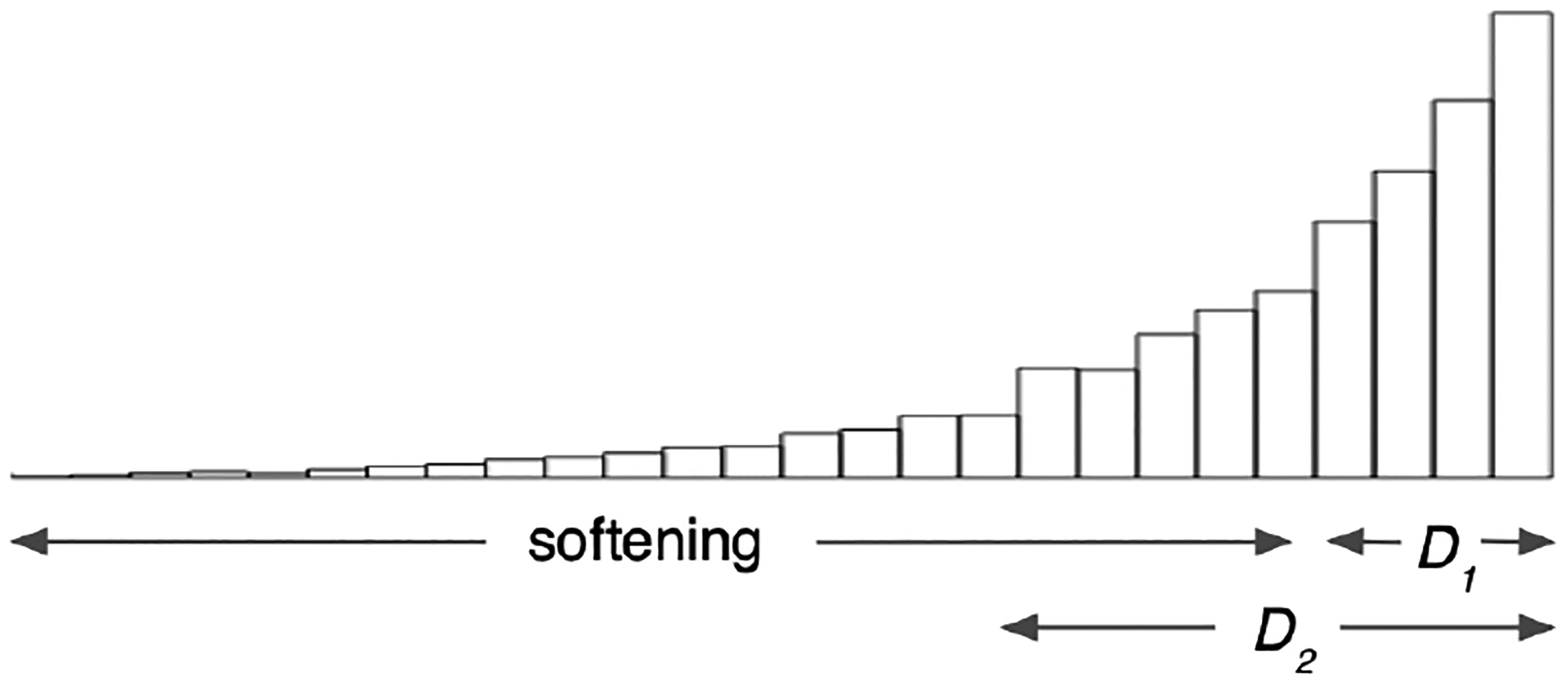
Hypothetical histogram of the singular values of a matrix. A rank *D*_1_ PARAFAC approximation incorporates the components which correspond to the *D*_1_ largest singular values. Increasing the rank to *D*_2_ allows for *D*_2_ − *D*_1_ additional components, whereas Softer allows for deviations corresponding to any singular value.

However, if the true coefficient tensor is not of low rank, Softer can expand away from a low-rank structure in two ways: it can use additional components in the underlying hard PARAFAC, or it can soften away from the rigid underlying low-rank structure. The deviations based on the PARAFAC softening can effectively capture components corresponding to singular values of any magnitude. In Figure 4 we illustrate the range of singular values that would be accommodated when expanding away from a rank-*D*_1_ hard PARAFAC approximation by (1) increasing the hard PARAFAC rank, and (2) softening the PARAFAC. Increasing the hard PARAFAC rank would include components corresponding to some small singular values, but softening the PARAFAC accommodates deviations from the underlying *D*_1_-rank structure across all singular values. Since Softer based on an underlying *D*-rank can capture these arbitrary deviations from a *D*-rank structure, increasing the rank *D* in Softer’s underlying PARAFAC is not expected to drastically alter the results, making Softer *robust* to the choice of rank *D*. This intuition is empirically evaluated in Section 5.2.

Softer’s robustness to the choice of the underlying rank *D* and its ability to capture *any* true coefficient tensor is also evident in the following theoretical results. In Proposition 3 we show that the prior on ***B***, *π*_***B***_, has full *L*_∞_ prior support in that it assigns positive prior weight to any *ϵ*-sized neighborhood of any true coefficient tensor ***B***^0^. This indicates that, even if the value of *D* is lower than the coefficient tensor’s true rank, Softer assigns positive prior weight to a neighborhood of the true tensor. Full prior support is a key element for establishing sufficient flexibility of a Bayesian procedure. In turn, the posterior distribution of the coefficient tensor is consistent, irrespective of the true coefficient tensor’s rank, or the rank used in Softer’s underlying PARAFAC.

**Proposition 3 (Full prior support)** *Let ϵ* > 0*. Then*, $\pi_{B}\left(B_{\epsilon }^{\infty }\left(B^{0}\right)\right)>0$, *where* $B_{\epsilon }^{\infty }\left(B^{0}\right)=\left\{B:{\text{max}}_{\underset{˜}{j}}\left|B_{\underset{˜}{j}}^{0}-B_{\underset{˜}{j}}\right|<\epsilon \right\}$.

We assume that the true data generating model is Equation 2 with true coefficient tensor ***B***^0^, and that the tensor predictor ***X*** has bounded entries. Since our interest is in estimating ***B***^0^, we assume that *τ*^2^ = 1, *μ* = 0 and ***δ*** = **0** are known. Then, we have the following.

**Proposition 4** *The posterior distribution of* ***B*** *is weakly consistent for* ***B***^0^.

These results indicate that softening the PARAFAC allows us to capture any true coefficient tensor. Since they hold irrespective of the choice of the underlying rank, they also indicate that Softer is robust to the choice of rank *D*, at least asymptotically.

### 4.6 Approximating the Posterior Distribution of the Coefficient Tensor

We approximate the posterior distribution of ***B*** using Markov chain Monte Carlo (MCMC). An MCMC scheme where most parameters are updated using Gibbs sampling is shown in Appendix C. We found this approach to be sufficiently efficient when the sample size is larger or of similar order to the number of parameters. However, in very high-dimensional settings where *p* ≫ *n*, mixing and convergence was slow under reasonable time constraints. For that reason, and in order to provide a sampling approach that performs well across *n*, *p* situations, we instead rely on Hamiltonian Monte Carlo (HMC) implemented in Stan (Carpenter et al., 2017) and on the R interface (Stan Development Team, 2018) to acquire samples from the posterior distribution. HMC is designed to improve mixing relative to Gibbs sampling by employing simultaneous updates, and relying on gradients calculated with automatic differentiation to obtain efficient proposals. Using Stan, we employ the No-U-Turn sampler (NUTS) algorithm (Hoffman and Gelman, 2014) which automatically tunes the HMC parameters to achieve a target acceptance rate (the default step size adaptation parameters were used). If MCMC convergence is slow, one could increase the NUTS parameter *δ* in RStan from 0.8, which is the default, to a value closer to 1.

MCMC convergence was assessed based on visual inspection of traceplots across chains with different starting values and the potential scale reduction factor (Gelman and Rubin, 1992) for the regression coefficients *μ*, ***δ***, ***B*** and the residual variance *τ*^2^. Note that the remaining parameters are not individually identifiable as the underlying PARAFAC parameters are themselves non-identifiable, and therefore the corresponding softening parameters are also non-identifiable. In simulations, we considered a model that restricts the underlying PARAFAC parameters to make them identifiable (using the constraints discussed in Section 2.3 of Guhaniyogi et al. (2017)). We found that the original and constrained models had identical estimation performance, but that the MCMC for the unconstrained model including non-identifiable parameters required a smaller number of iterations to converge than the constrained model that uses the identifiable underlying PARAFAC.

## 5. Simulations

To illustrate the performance of Softer and compare it against alternatives, we simulated data under various scenarios. In one set of simulations (Section 5.1), we considered a tensor predictor of dimension 32 × 32, corresponding coefficient tensors ranging from close to low-rank to full-rank, and with different degrees of sparsity, and sample size equal to 400. In another set of simulations (Section 5.2), we considered a tensor predictor of dimension 20 × 20 and corresponding coefficient tensor of rank 3, 5, 7, 10 and 20 in order to investigate the performance of Softer relative to the hard PARAFAC for a true coefficient tensor that increasingly deviates from low rank form, and various choices of the algorithmic rank *D*. The sample size in this situation was 200. In all scenarios, the predictor’s entries were drawn independently from a *N*(0, 1) distribution, and the outcome was generated from a model in the form Equation 2 with true residual variance *τ*^2^ = 0.5.

We considered the following methods: (a) Softer, (b) the Bayesian hard PARAFAC approach of Guhaniyogi et al. (2017), and (c) estimating the coefficient tensor by vectorizing the predictor and performing Lasso. We considered these two competing approaches because they represent the two extremes of how much prioritization is given to the predictor’s array structure (the hard PARAFAC directly depends on it, the Lasso completely ignores it), whereas Softer is designed to exploit the predictor’s structure while allowing deviations from it. We also considered (d) the Bayesian tensor regression approach of Spencer (2020) which is based on the Tucker decomposition, a generalization of the PARAFAC decomposition.

Methods were evaluated in terms of how well they estimated the true coefficient tensor ***B*** by calculating (1) the entry-specific bias and mean squared error of the posterior mean (for the Bayesian approaches) and the penalized likelihood estimate (for the Lasso), and (2) the frequentist coverage of the 95% credible intervals. In order to evaluate the methods’ performance in accurately identifying important entries (entries with non-zero coefficients), we calculated methods’ (3a) sensitivity (percentage of important entries that were identified), (3b) specificity (percentage of non-important entries that were correctly deemed non-important), (3c) false positive rate (percentage of identified entries that are truly not important), and (3d) false negative rates (percentage of non-identified entries that are important). For the Bayesian methods, an entry was flagged as important if its corresponding 95% credible interval did not overlap with zero. Hierarchical Bayesian models have been shown to automatically perform adjustment for multiple testing error (Scott and Berger, 2010; Müller et al., 2006). Confidence intervals and entry selection for the Lasso were not considered. We also evaluated the models’ predictive performance using (4) the predictive mean squared error defined as the mean of the squared difference between the true outcome and the predictive mean over 1,000 new data points. Lastly, (5) we compared the hard PARAFAC and Softer in terms of their computational time, for various ranks *D*.

Additional simulations are shown in Appendix D and are summarized below, where applicable.

### 5.1 Simulation Results for Tensor Predictor of Dimension 32×32

**Figure 5:**
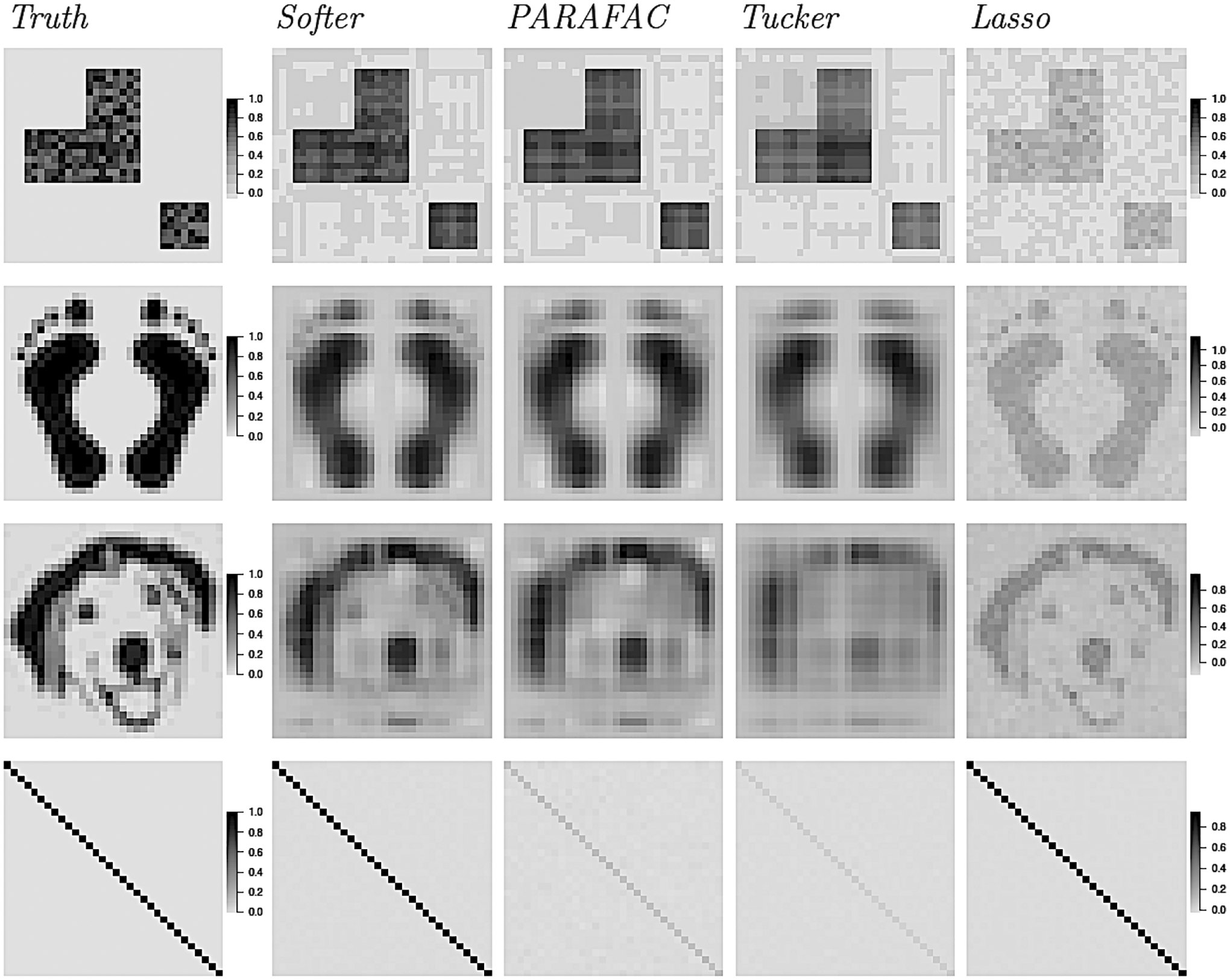
True and average estimated coefficient matrix based on Softer, hard PARAFAC, Tucker regression, and Lasso.

The scenarios we considered represent settings of varying complexity and sparsity. The first column of Figure 5 shows the true coefficient tensors (squares, feet, dog, diagonal). The squares coefficient matrix is used as a scenario where the true coefficient matrix is rectangular, but not low rank, and not sparse. The next two scenarios represent situations where the underlying structure is not of low-rank form, but could be potentially approximated by a low rank matrix up to a certain degree, hence representing scenarios somewhat favorable to the hard PARAFAC. In the last case, the diagonal coefficient matrix is used to represent a sparse coefficient matrix of full-rank without a network-structure, a scenario that is favorable for the Lasso, but is expected to be difficult for the hard PARAFAC. Therefore, the scenarios we consider here represent a wide range of rank and sparsity specifications, and we investigate Softer’s ability to use a low rank structure when such structure is useful (feet, dog), and expand away from it when it is not (squares, diagonal). Even though none of these coefficient tensors is low-rank, we considered a scenario with a low-rank coefficient matrix in the Appendix, and we discuss it briefly below.

The remaining columns of Figure 5 show the average posterior mean or penalized estimate across simulated data sets. Results from Softer and the hard PARAFAC correspond to *D* = 3, though the methods are also considered with rank *D* = 7 (the results are shown in the Appendix and are discussed below). In the squares, feet and dog scenarios, the hard PARAFAC performs decently in providing a low-rank approximation to the true coefficient matrix. However, certain coefficient entries are estimated poorly to fit its rectangular structure. This is most evident in the squares scenario where the non-zero coefficients are obscured in order to fit in a low-rank form. Results for the approach based on the Tucker decomposition are worse, with estimated coefficient matrices that deviate from the truth more. In the diagonal scenario, the hard PARAFAC and Tucker approaches totally miss the diagonal structure and estimate (on average) a coefficient matrix that is very close to zero. In contrast, the Lasso performs best in the sparse, diagonal scenario and estimates on average the correct coefficient matrix structure. However, in the squares, dog and feet settings, it underestimates the coefficient matrix since it is based on assumed sparsity which is not true, and does not borrow any information across coefficient matrix entries. In all situations, Softer closely identifies the structure of the underlying coefficient matrix, providing a compromise between tensor-based and unstructured estimation, and having small biases across all simulated scenarios (average bias also reported in Table 1). In the squares, feet and dog scenarios, Softer bases estimation on the low-rank structure of the underlying hard PARAFAC, but it expands from it to better describe the true coefficient matrix’s details. At the same time, Softer also performs well in the diagonal scenario, where the true coefficient tensor is full-rank. Therefore, the strength of Softer is found in its ability to use the low-rank structure of the PARAFAC when necessary, and diverge from it when needed.

In Table 1, we report the methods’ bias and root mean squared error (rMSE), predictive mean squared error, and frequentist coverage of the 95% credible intervals. Conclusions remain unchanged, with Softer performing similarly to the hard PARAFAC when its underlying structure is close to true, and has the ability to diverge from it and estimate a coefficient tensor that is not low-rank in other scenarios. This is evident by an average coverage of 95% posterior credible intervals that is 94.7% in the diagonal scenario. In terms of their predictive ability, the pattern observed for the bias and mean squared error persists, with Softer having the smallest mean squared predictive error in the squares, feet and dog scenarios, and the Lasso for the diagonal scenario, followed closely by Softer. Across all scenarios, the approach based on the Tucker decomposition performs worst among the Bayesian methods in terms of both estimation and predictive accuracy.

Table 2 shows the performance of Softer, the hard PARAFAC, and Tucker regression approaches for identifying the important entries of the tensor predictor (entries with non-zero coefficients). Perfect performance would imply specificity and sensitivity equal to 100, and false positive and negative rates (FPR, FNR) equal to 0. The methods perform comparably in terms of specificity, sensitivity, and FNR, except for the diagonal scenario where the sensitivity of the hard PARAFAC and Tucker approaches is dramatically lower. However, the big difference is in the FPR. Even though Softer’s FPR is at times higher than 5%, it remains at much lower levels than the hard PARAFAC and Tucker approaches for which FPR is on average over 10% in the dog and approximately 30% in the diagonal scenario. These results indicate that identifying important entries based on the hard PARAFAC could lead to overly high false discovery rates, whereas Softer alleviates this issue. In Appendix D.1 we investigate the cases where Softer and hard PARAFAC return contradicting results related to an entry’s importance. We illustrate that, when PARAFAC identifies entries as important and Softer does not, it is most often entries with small coefficient values, in an effort to fit the coefficient matrix into a low-rank form.

Appendix D shows additional simulation results. Appendix D.2 shows results for alternative coefficient matrices, including a coefficient matrix of rank 3, a scenario favorable to the hard PARAFAC. There, we see that Softer collapses to the underlying low-rank structure when such a structure is true. These simulation results are in agreement with our first intuition-based argument of Section 4.5 with regards to the choice of rank *D* for Softer. Appendix D.3 shows results for a subset of the coefficient matrices and for sample size *n* = 200.

Simulations with a smaller *n* to *p* ratio show that Softer performs comparably to the hard PARAFAC for the dog and feet scenarios and has substantially smaller bias and rMSE for the truly non-zero coefficients in the diagonal scenario. The most notable conclusion is that Softer results are closer to the hard PARAFAC results when the sample size is small. This indicates that the data inform the amount of PARAFAC softening which depends on the sample size. Finally, Appendix D.4 shows results for Softer and the hard PARAFAC when *D* = 7. Even though the hard PARAFAC shows substantial improvements using the higher rank, Softer performs almost identically for rank 3 or 7, illustrating its robustness to the choice of the underlying rank. This last conclusion is further investigated in Section 5.2.

### 5.2 Simulation Results for Coefficient Tensor of Increasing Rank

We evaluated the performance of the hard and soft PARAFAC tensor regression with various values of the algorithmic rank *D* and the coefficient tensor’s true rank. We considered tensor predictor of dimension 20 × 20, rank of the true coefficient matrix equal to 3, 5, 7, 10 and 20, and we evaluated the hard PARAFAC and Softer with *D* ∈ {1, 3, 5, 7, 10}. For every value of the true rank, we generated 100 data sets.

**Figure 6:**
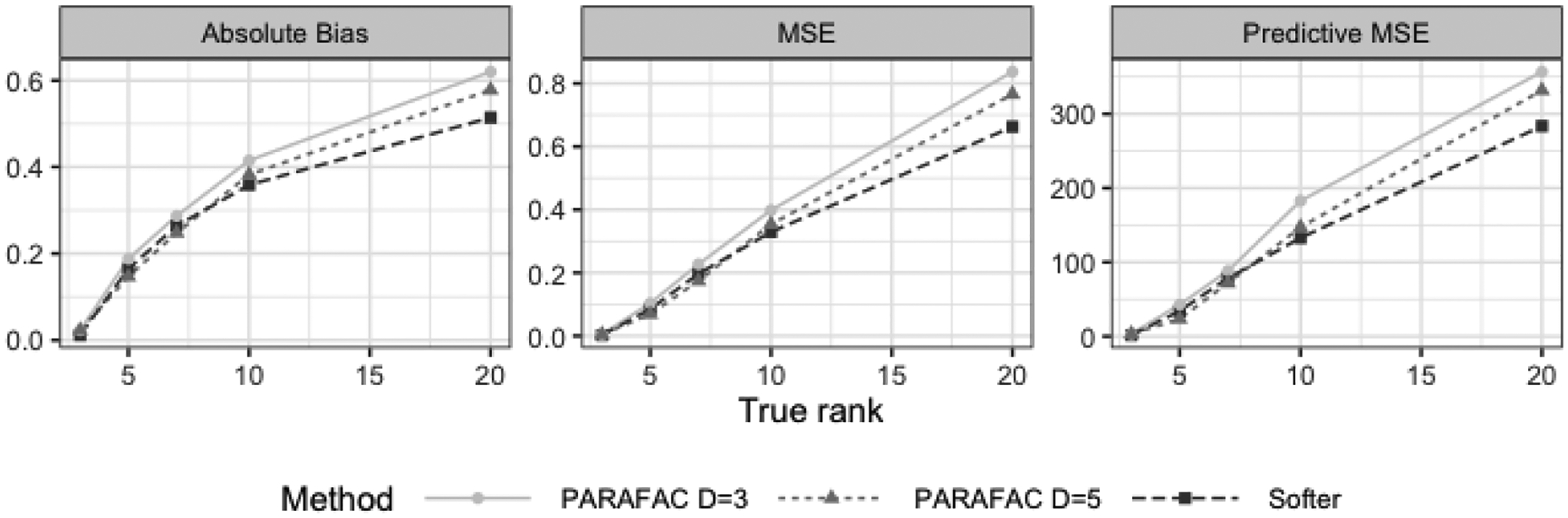
Average absolute bias, estimation mean squared error and predictive mean squared error (y-axis) for tensor predictor of dimensions 20 × 20 and true coefficient matrix of increasing rank (x-axis). Results are shown for the hard PARAFAC with *D* = 3 and *D* = 5, and Softer with *D* = 3.

In Figure 6, we show the average across entries of the coefficient matrix of the absolute bias and mean squared error, and the predictive mean squared error. For illustrative simplicity, we include a subset of the results: Softer with *D* = 3, and the hard PARAFAC with *D* = 3 and *D* = 5, though the complete results are included in the Appendix and discussed below. When the true rank of the coefficient matrix is 3, the three approaches perform similarly. This indicates that both the hard PARAFAC with *D* = 5 and Softer are able to convert back to low ranks when this is true. For true rank equal to 5 or 7, the hard PARAFAC with *D* = 5 slightly outperforms Softer. However, for *D* > 7, Softer based on a rank-3 underlying structure performs best both in estimation and in prediction. These results indicate that, in realistic situations where the coefficient tensor is not of low-rank form, Softer with a low rank has the ability to capture the coefficient tensor’s complex structure more accurately than the hard PARAFAC.

In Appendix D.5, we show the performance of Softer and the hard PARAFAC across all values of *D* considered, *D* ∈ {1, 3, 5,7, 10}. The conclusions about the methods’ relative performance remain unchanged: even though Softer and the hard PARAFAC perform similarly for coefficient tensors with small true rank, Softer with *D* > 1 outperforms the hard PARAFAC in terms of both estimation and prediction when the coefficient tensor is of high rank. These results illustrate that the performance of the hard PARAFAC depends heavily on the choice of rank *D*. In contrast, Softer’s performance is strikingly similar across all values of the algorithmic rank, supporting the second intuition-based argument on Softer’s robustness in Section 4.5.

Finally, Table 3 shows the average time across simulated data sets for 15,000 MCMC iterations for the two methods. As expected, the computational time for both approaches increases as the value of *D* increases, though the true rank of the coefficient tensor seems to not play a role. Here, we see that Softer generally requires two to three times as much time as the hard PARAFAC *for the same value of D*, which might seem computationally prohibitive at first. However, our results in Figure 6 and Appendix D.5 show that Softer requires *a smaller value of D* in order to perform similarly to or better than the hard PARAFAC in terms of both estimation and prediction. Therefore, Softer’s computational burden can be drastically alleviated by fitting the method for a value of *D* which is substantially lower than the value of *D* for the hard PARAFAC. In Section 7, we also discuss frequentist counterparts to our model that might also reduce the computational load.

## 6. Estimating the Relationship Between Brain Connectomics and Human Traits

Data from the Human Connectome Project (HCP) contain information on about 1,200 healthy young adults including age, gender, various brain imaging data, and a collection of measures assessing cognition, personality, substance intake and so on (referred to as traits). We are interested in studying the brain structural connectome, referring to anatomical connections of brain regions via white matter fibers tracts. The white matter fiber tracts can be indirectly inferred from diffusion MRI data. Two brain regions are considered connected if there is at least one fiber tract running between them. However, there can be thousands of fiber tracts connecting a pair of regions. Properties of the white matter tracts in a connection, such as number of tracts, and patterns of entering the regions, might be informative about an individual’s traits. Using data from the HCP and based on the soft tensor regression framework, we investigate the relationship between different connectome descriptors and human traits.

Structural connectivity data were extracted using state-of-the-art pipelines in Zhang et al. (2018). In total, about 20 connectome descriptors (adjacency matrices) describing different aspects of white matter fiber tract connections were generated (see Zhang et al. (2018) for more information on the extracted descriptors). Each adjacency matrix has a dimension of 68 × 68, representing *R* = 68 regions’ connection pattern. The 68 regions were defined using the Desikan-Killiany atlas (Desikan et al., 2006). Of the 20 extracted connectome features, we consider two in this analysis: (a) count, describing the number of streamlines, and (b) connected surface area (CSA), describing the area covered by small circles at the interactions of fiber tracts and brain regions, since they are the most predictive features according to results in Zhang et al. (2019).

We examine the relationship between these descriptors of structural brain connections and 15 traits, covering domains such as cognition, motor, substance intake, psychiatric and life function, emotion, personality and health. The full list of outcomes we analyze is presented in Table E.1 and includes both binary and continuous traits. For binary traits, a logistic link function is assumed.

### 6.1 Adapting Softer for (Semi-)Symmetric Brain Connectomics Analysis

The nature of the brain connectivity data implies that the *R* × *R*-dimensional tensor predictor including a specific connectivity feature among *R* regions of interest (ROIs) is symmetric and the diagonal elements can be ignored since self-loops are not considered. Further, considering *p* features simultaneously would lead to an *R* × *R* × *p* tensor predictor which is semi-symmetric (symmetric along its first two modes). The (semi-)symmetry encountered in the predictor allows us to slightly modify Softer and reduce the number of parameters by imposing that the estimated coefficient matrix ***B*** is also (semi-)symmetric. We provide the technical details for the (semi-)symmetric Softer in Appendix F.

### 6.2 Analyses of the Brain Connectomics Data

For the purpose of this paper, we investigate the relationship between features of brain connections and human traits by regressing each outcome on each of the two predictors (count and CSA) separately. Even though analyzing the relationship between the traits and multiple features simultaneously is possible, we avoid doing so here for simplicity. We analyze the data employing the following methods: (1) symmetric Softer with *D* = 6, (2) the hard PARAFAC approach of Guhaniyogi et al. (2017) which does not impose symmetry of the coefficient tensor with *D* = 10, and (3) Lasso on the vectorized lower triangular part of the tensor predictor. Since publicly available code for non-continuous outcomes is not available for the hard PARAFAC approach, we only consider it when predicting continuous outcomes.

We compare methods relative to their predictive performance. For each approach, we estimate the out-of-sample prediction error by performing 15-fold cross validation, fitting the method on 90% of the data and predicting the outcome on the remaining 10%. In the case of Softer and the hard PARAFAC we also investigate the presence of specific brain connections that are important in predicting any of the outcomes by checking whether their coefficients’ 95% posterior credible intervals include zero. Additional results based on Softer for a different choice of baseline rank or when symmetry is ignored are included in Appendix E and are summarized below.

### 6.3 Using Features of Brain Connections for Predicting Human Traits

For continuous outcomes, the methods’ predictive performance was evaluated by calculating the percentage of the marginal variance explained by the model defined as 1 − (CV MSE)/(marginal variance). For binary outcomes, we used the model’s estimated linear predictor to estimate the optimal cutoff for classification based on Youden’s index (Youden, 1950) and calculated the average percentage of correctly classified observations in the held-out data.

**Figure 7:**
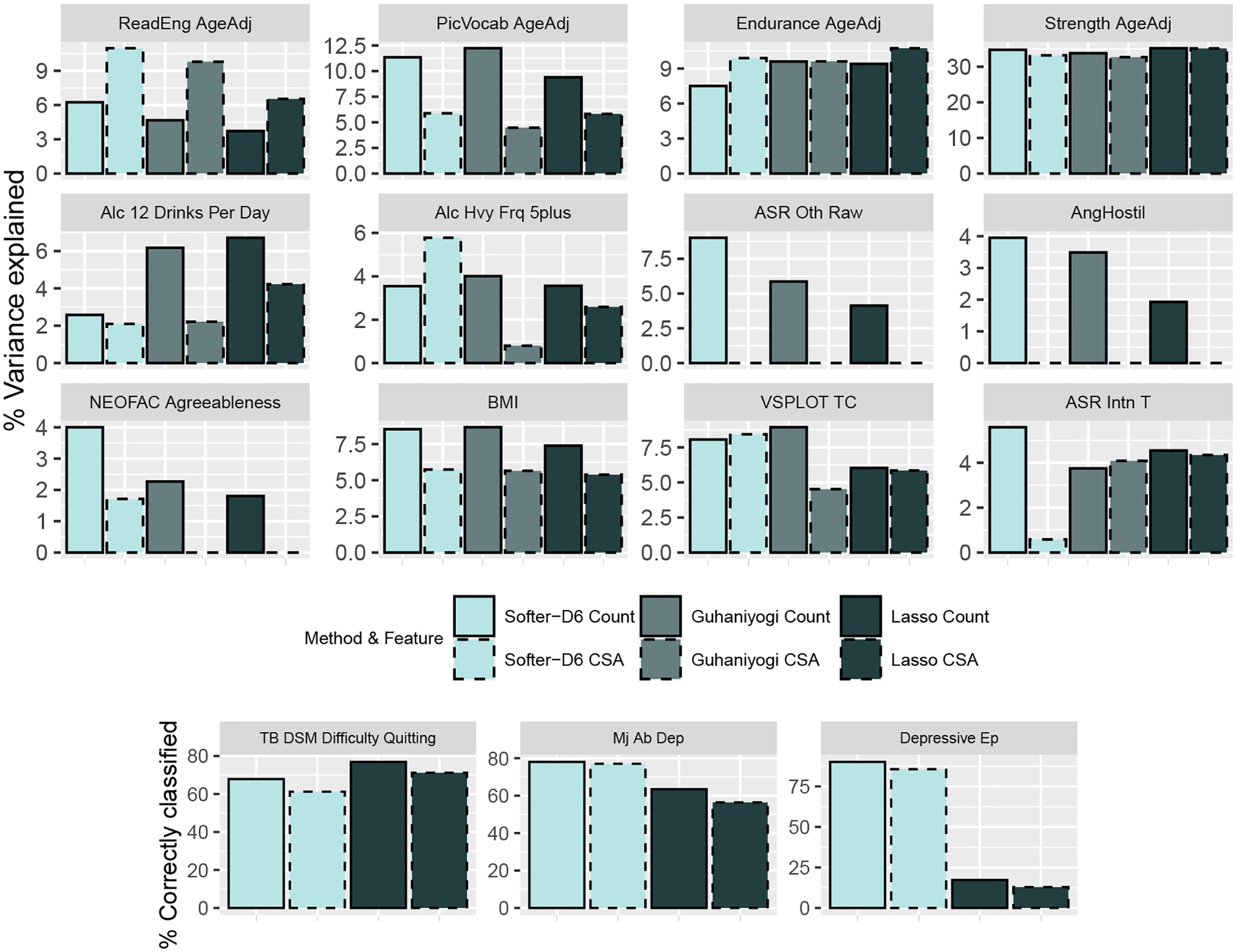
Top: Percentage of outcome variance explained by the tensor predictor for continuous outcomes calculated as [1 − MSE / (marginal variance)] ×100. Bottom: Average percentage of units correctly classified for binary outcomes. Results are presented using different color for each method, and different line-type for each feature of brain connections.

Figure 7 shows the results for the three approaches considered, and for each feature separately. For most outcomes, one of the two features appeared to be most predictive of the outcome across approaches. For example, the count of streamlines was more predictive than CSA of an individual’s anger level (AngHostil), independent of the modeling approach used. By examining the methods’ predictive performance, it is evident that features of brain connectomics are, in some cases, highly predictive of outcomes. Specifically, over 30% of the variance in an individual’s strength level, and over 10% of the variance in endurance, reading comprehension, and picture vocabulary ability can be explained by the count or CSA of streamlines of their brain connections.

Not one approach outperformed the others in prediction across all features and outcomes. However, approaches that accommodate the network structure perform better than Lasso in most situations. One example is Softer’s performance relative to Lasso when predicting individuals’ previous depressive episode (Depressive Ep). Here, Lasso performs worse than the random classifier, whereas Softer has over 90% accuracy. Even when the number of observations is less than 300 (difficulty quitting tobacco, TB DSM Difficulty Quitting), Softer performs only slightly worse than Lasso. For continuous outcomes, Softer and hard PARAFAC perform comparably. As we saw in the simulations in Section 5 and in Appendix D.3, the similar predictive performance of Softer and hard PARAFAC could be due to the limited sample size that forces Softer to heavily rely on the underlying low-rank structure for estimation, essentially reverting back to the hard PARAFAC.

The low signal in predicting some outcomes implies low power in identifying pairs of brain regions whose connection’s features are important. In fact, 95% credible intervals for all coefficients using the hard PARAFAC overlapped with zero. In contrast, Softer identified seven important connections: five of them were for predicting whether an individual has had a depressive episode (three using count of streamlines as the predictor, and two using CSA), one in predicting an individual’s strength, and one in predicting the variable short pennline orientation (VSPLOT). The identified connections are listed in Table 4 and agree with the literature in neuroscience. All identified connections in predicting a depressive episode involve the parahippocampal, which is the posterior limit of the amygdala and hippocampus and is located in the temporal lobe, and ROIs located in the frontal lobe (paracentral, lateral orbitofrontal, pars orbitalis). Dysfunction of the parahippocampal (as well as the amygdala and hippocampus) has been identified in various studies as an important factor in major depression and emotion-related memory observed in depression (Mayberg, 2003; Seminowicz et al., 2004; LaBar and Cabeza, 2006; Zeng et al., 2012). Further, dysregulation of the pathways between the frontal and temporal lobes has been identified as predictive of depression (Mayberg, 1994; Steingard et al., 2002), even when explicitly focusing on the cortical regions Softer identified as important (Liao et al., 2013). Two of the three connections were identified as important irrespective of the tensor predictor used (count or CSA). Even though these predictors are related, they describe different aspects of brain connectivity. Therefore, Softer identified the same connections as important based on two separate measures of brain connectivity. The identified connection in predicting strength involves the precuneus and superior parietal regions in the parietal lobe. Precuneus’ connectivity has been associated with a variety of human functions, including motor-related traits (Cavanna and Trimble, 2006; Wenderoth et al., 2005; Simon et al., 2002), and the parietal lobe in general is believed to control humans’ motor system (Fogassi and Luppino, 2005).

Appendix E.2 includes results from the symmetric Softer using a smaller rank (*D* = 3), and from Softer using the same rank as the results in this section (*D* = 6) but ignoring the known symmetry of the predictor. All three versions of Softer perform similarly in terms of prediction, with potentially slightly lower predictive power for the symmetric Softer with rank 3. The entries identified as important by Softer with *D* = 3 are similar, though not identical to the ones in Table 4. When symmetry is not accounted for, Softer does not identify any important connections. Finally, in Appendix E.3 we investigate the reproducibility of identified connections across random subsamples including 90% of the observations. We find that for strength, when connections are identified in the subsample, they generally include the ones in Table 4. Finally, when predicting whether an individual has has a depressive episode, *any* of the connections identified across *any* subsample and for *either* predictor involved at least one of Parahippocampal the (in the left hemisphere) or Lateral Orbitofrontal (in the right hemisphere) ROIs, implying that the importance of these two regions is strikingly reproducible.

## 7. Discussion

In this paper, we considered modeling a scalar outcome as a function of a tensor predictor within a regression framework. Estimation of regression models in high dimensions is generally based on some type of assumed sparsity of the true underlying model: sparsity directly on covariates, or “latent sparsity” by assuming a low-dimensional structure. When the assumed sparsity is not true, the model’s predictive ability and estimation can suffer. Our approach is positioned within the class of latent sparsity, since it exploits a low-dimensional underlying structure. We focused on adequately relaxing the assumed structure by softening the low-dimensional PARAFAC approximation and allowing for interpretable deviations of row-specific contributions. We show that softening the PARAFAC leads to improved estimation of coefficient tensors, better performance in identifying important entries, consistent estimation irrespective of the underlying rank used for estimation, and more accurate predictions. The approach is applicable to both continuous and binary outcomes, and was adapted to (semi-)symmetric tensor predictors, which is common in settings where the predictor is measured on a network of nodes. Softer was used to study the relationship between brain connectomics and human traits, and identified several important connections in predicting depression.

Combining the two types of assumed sparsity for low-rank *and* sparse matrix estimation has received some attention in the literature, especially in machine learning with matrix data. Candès et al. (2011), Waters et al. (2011) and Zhou and Tao (2011) decomposed matrices as the sum of a low-rank and a sparse component. Zhang et al. (2016) employed such decomposition in anomaly detection by studying the Mahalanobis distance between the observed data and the low-rank component. Richard et al. (2012) developed a penalization-based approach to estimating matrices that are simultaneously sparse and low-rank by adopting one type of penalty for sparsity and one for rank. All these approaches have been formulated as optimization problems and algorithms are generally based on iterative procedures.

Within a Bayesian regression framework, Guha and Rodriguez (2021) combined the two types of sparsity and proposed a network-based spike and slab prior on the nodes’ importance. Under that model, a node is either active or inactive, and active nodes are expected to contribute to the outcome based on a low-rank coefficient tensor. In that sense, this approach has important commonalities to estimating a coefficient tensor that is simultaneously sparse and low-rank. Even though we find that approach to be promising, we find that node selection in itself can be too restrictive in some settings, and future work could incorporate hierarchical or parallel node and entry selection.

Softer has similarities but also differences from the methods discussed above. On one hand, Softer provides a relaxation of an assumed low-rank form. However, this relaxation (or softening) is not sparse in any way, and *every* element of the tensor is allowed to deviate from the low-rank structure. We find that an exciting line of research would combine low-rank and sparse approaches while allowing for sufficient flexibility to deviate from both of them. Important questions remain on the interplay between entry selection and the assumed structure on the coefficient tensor. We illustrated in simulations that tensor regression based on the hard PARAFAC can perform poorly for entry selection. Future work could focus on studying the properties of multiplicity control in structured settings, and forming principled variable selection approaches with desirable properties within the context of structured data.

Even though we showed that the posterior distribution of the coefficient tensor based on Softer is consistent irrespective of the tensor’s true rank or the algorithmic rank used, additional theoretical results would help illuminate Softer’s performance. For example, it would be interesting to understand how Softer performs under the asymptotic regime where the tensor predictor is allowed to grow with the sample size. Furthermore, even though our simulation results indicate that Softer reverts back to the hard PARAFAC when the underlying low-rank structure is true, future approaches to “soft” regression can investigate whether the soft and hard approaches asymptotically converge if the hard structure is true. If this result was derived, then it would imply a new form of robustness: robustness in terms of the method chosen, soft or hard.

Finally, a criticism of Bayesian methods is often their computational burden. One way forward in alleviating this issue for Softer is to re-formulate our model within the frequentist paradigm. For example, one could impose a penalty term on the magnitude of the $\gamma_{k,j_{k}}^{\left(d\right)}$ parameters using ${\text{penalty}}_{\gamma }(d)=\sum_{k}\sum_{j_{k}}{\left(\gamma_{k,j_{k}}^{\left(d\right)}\right)}^{2}$, substitute the softening structure in Equation 6 with a penalty term such as ${\text{penalty}}_{\beta }(d)=\sum_{k}\sum_{\underset{˜}{j}}{\left(\beta_{k,\underset{˜}{j}}^{\left(d\right)}-\gamma_{k,j_{k}}^{\left(d\right)}\right)}^{2}$, and maximize the penalized likelihood with appropriate tuning parameters. One might need to link the tuning parameter for penalty_*γ*_(*d*) with that of penalty_*β*_(*d*) to ensure that penalization of one does not lead to overcompensation from the other (similarly to our discussion in Section 4.3 for the inclusion of *ζ*^(*d*)^ in the distribution for $\beta_{k,\underset{˜}{j}}^{\left(d\right)}$. Such *L*2-norm penalties with a continuous outcome would imply that the maximization problem is convex and optimization procedures can be developed relatively easily. Even though this reformulation would not reduce the number of parameters, it is expected to require far fewer iterations than an MCMC procedure. Alternative norms could also be considered to impose more sparsity, such as using a group-Lasso penalty instead of penalty_*β*_(*d*).

## Figures and Tables

**Table 1:** Simulation Results. Average bias, root mean squared error, frequentist coverage of 95% credible intervals among truly zero and truly non-zero coefficient entries, and predictive mean squared error for Softer (with *D* = 3), the hard PARAFAC (with *D* = 3), Tucker regression, and Lasso for the simulation scenario with tensor predictor of dimensions 32 × 32 and sample size *n* = 400. Bold text is used for the approach performing best in each scenario and for each metric. If no entry is bold, no conclusive argument can be made.

			Softer	PARAFAC	Tucker	Lasso
squares	Truly zero	bias	**0.003**	0.005	0.013	0.012
		rMSE	**0.033**	0.05	0.05	0.143
		coverage	99.7%	98.3%	99.8%	–
	Truly non-zero	bias	**0.084**	0.106	0.136	0.501
		rMSE	**0.11**	0.148	0.166	0.601
		coverage	80.1%	68.4%	**90.7%**	–
	Prediction	MSE	**5.05**	8.99	12.16	111.8
feet	Truly zero	bias	0.037	0.046	0.057	**0.016**
		rMSE	**0.092**	0.109	0.102	0.198
		coverage	96.9%	94.2%	95.5%	–
	Truly non-zero	bias	**0.116**	0.138	0.175	0.43
		rMSE	**0.184**	0.21	0.226	0.558
		coverage	**89.2%**	80%	84.8%	–
	Prediction	MSE	**31.9**	41.8	50.6	264.6
dog	Truly zero	bias	0.047	0.059	0.083	**0.029**
		rMSE	**0.111**	0.129	0.129	0.151
		coverage	98.0%	92.8%	95.5%	–
	Truly non-zero	bias	**0.129**	0.159	0.214	0.350
		rMSE	**0.205**	0.230	0.261	0.435
		coverage	**88.2%**	76.9%	78.5%	–
	Prediction	MSE	**35.0**	45.4	61.7	138.4
diagonal	Truly zero	bias	0.002	0.004	0.002	<**0.001**
		rMSE	0.02	0.051	0.024	**0.009**
		coverage	100%	100%	100%	–
	Truly non-zero	bias	0.111	0.899	0.954	**0.07**
		rMSE	0.126	0.906	0.955	**0.084**
		coverage	**94.7%**	3%	0.8%	–
	Prediction	MSE	1.41	29.7	30.6	**0.81**

**Table 2:** Methods’ performance in identifying important entries. For sensitivity, specificity and false negative rate (FNR), results are shown as average across simulated data sets (×100), and for false positive rate* (FPR) as average (10^*th*^, 90^*th*^ percentile) (×100)

		Sensitivity	Specificity	FPR	FNR
squares	Softer	100	99.7	0.9 (0, 2.5)	0
	PARAFAC	100	98.3	4.7 (1.2, 8.7)	0
	Tucker	100	99.9	0.3 (0, 0.4)	0
feet	Softer	64.5	96.9	2.9 (1.7, 4.1)	36.3
	PARAFAC	68.4	94.1	5.2 (3.3, 7.1)	34.3
	Tucker	63.6	95.5	4.4 (3.3, 5.5)	37.2
dog**	Softer	52.9	96.7	5.2 (2.7, 8.1)	34.7
	PARAFAC	63.1	90.1	12.4 (8.4, 15.9)	30.9
	Tucker	46.3	93.1	11.5 (5.2, 17.6)	38.6
diagonal	Softer	100	100	0 (0, 0)	0
	PARAFAC	3	100	28.8 (0, 70)	3
	Tucker	< 1	100	33.3 (10, 50)	3.1

* The average FPR is taken over simulated data sets for which at least one entry was identified as important.

** Most coefficients in the dog simulation were non-zero. Results are presented considering coefficients smaller than 0.05 as effectively zero.

**Table 3:** Computational time (in minutes) for Softer and the hard PARAFAC.

		True rank of coefficient tensor				
		3	5	7	10	20
Softer	*D* = 1	28	29	30	37	39
	*D* = 3	166	110	101	104	117
	*D* = 5	181	149	158	159	152
	*D* = 7	212	189	205	216	220
	*D* = 10	275	236	255	288	259
Hard PARAFAC	*D* = 1	16	16	15	15	15
	*D* = 3	30	39	46	35	36
	*D* = 5	69	54	57	53	56
	*D* = 7	100	90	82	72	76
	*D* = 10	104	117	108	104	102

**Table 4:** Brain connections with important features in predicting human traits.

Outcome	Feature	ROI 1	ROI 2
Depressive Episode	Count	(lh) Parahippocampal	(rh) Lateral Orbitofrontal
			(lh) Paracentral
			(lh) Pars Orbitalis
	CSA		(rh) Lateral Orbitofrontal
			(lh) Paracentral
Strength	Count	(rh) Precuneus	(rh) Superior Parietal
VSPLOT	CSA	(lh) Banks of Superior Temporal Sulcus	(lh) Rostral Anterior Cingulate

**Figure B.1:**
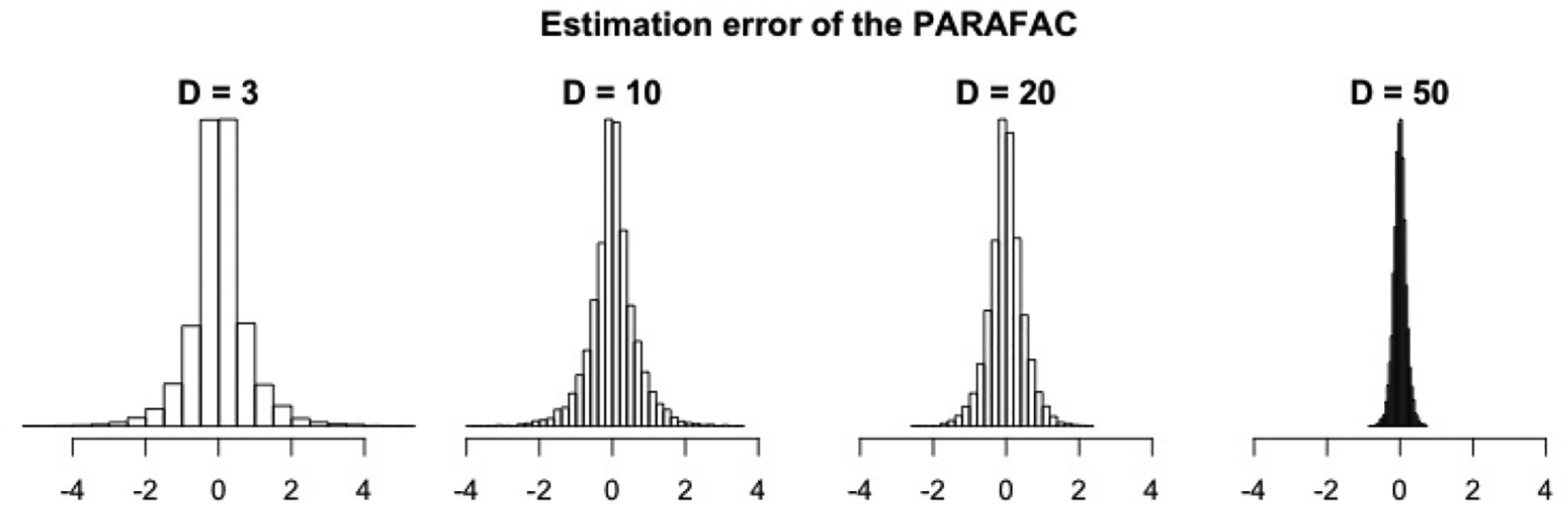
Distribution of errors based on the hard PARAFAC with increasing rank. The true matrix ***B*** resembles but is not exactly equal to a rank-1 tensor, and estimation is based on the singular value decomposition using *D* ∈ {3, 10, 20, 50} factors.

**Figure D.1:**
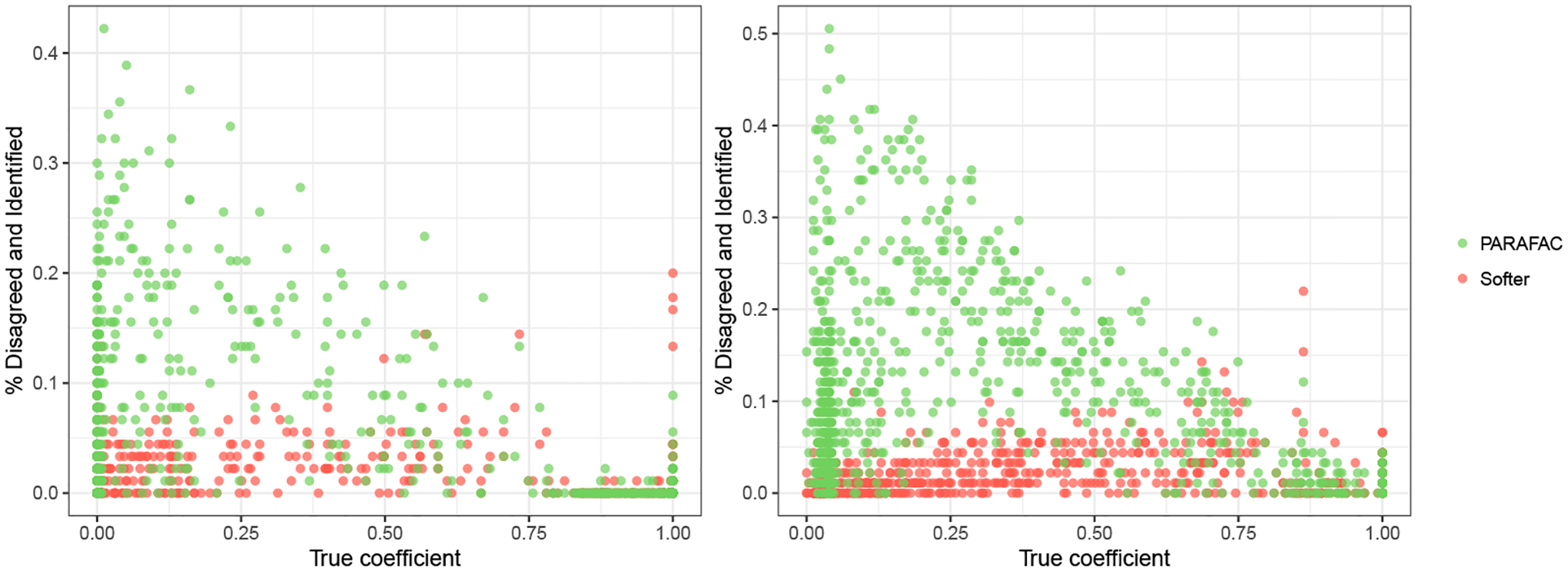
Percentage of simulated data sets that an entry with coefficient shown on the x-axis was identified as important by the stated method but not of the other one in the feet (left) and dog (right) scenario respectively.

**Figure D.2:**
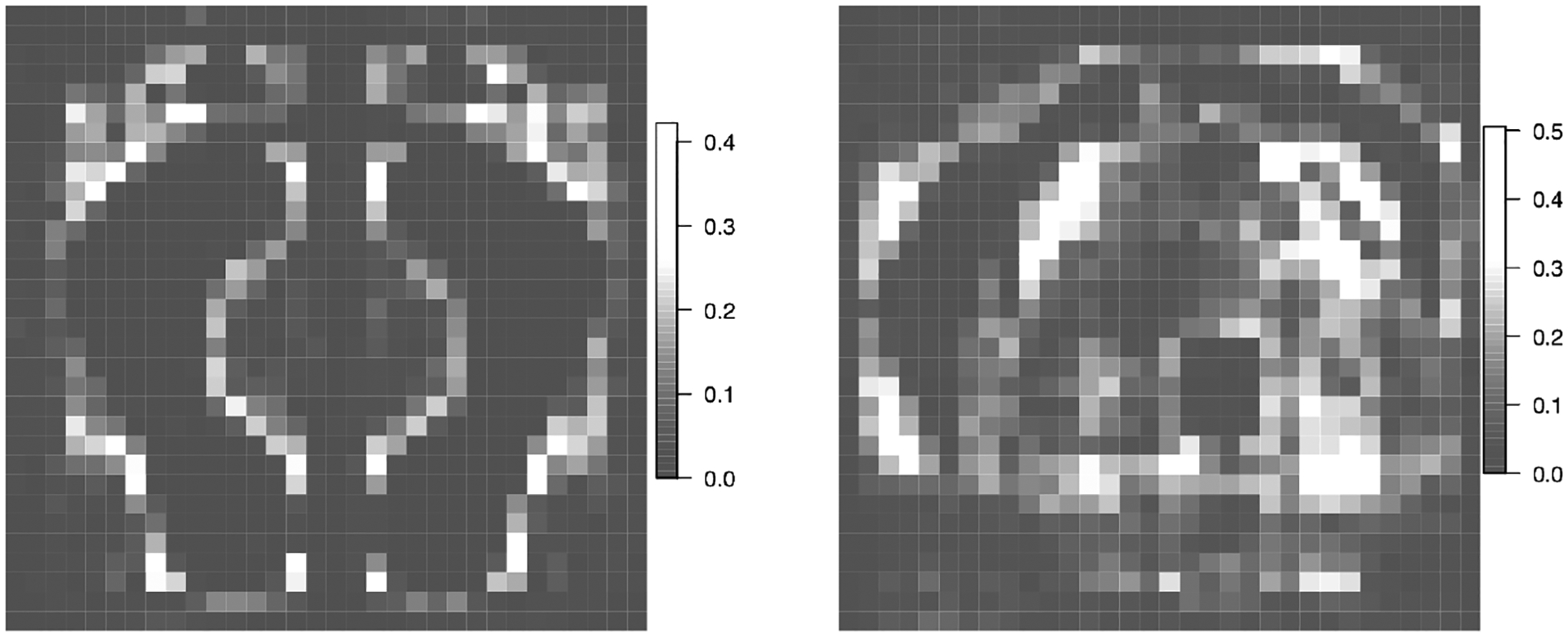
Percentage of data sets that an entry was identified as important by hard PARAFAC and not by Softer in the feet (left) and dog (right) scenario.

**Figure D.3:**
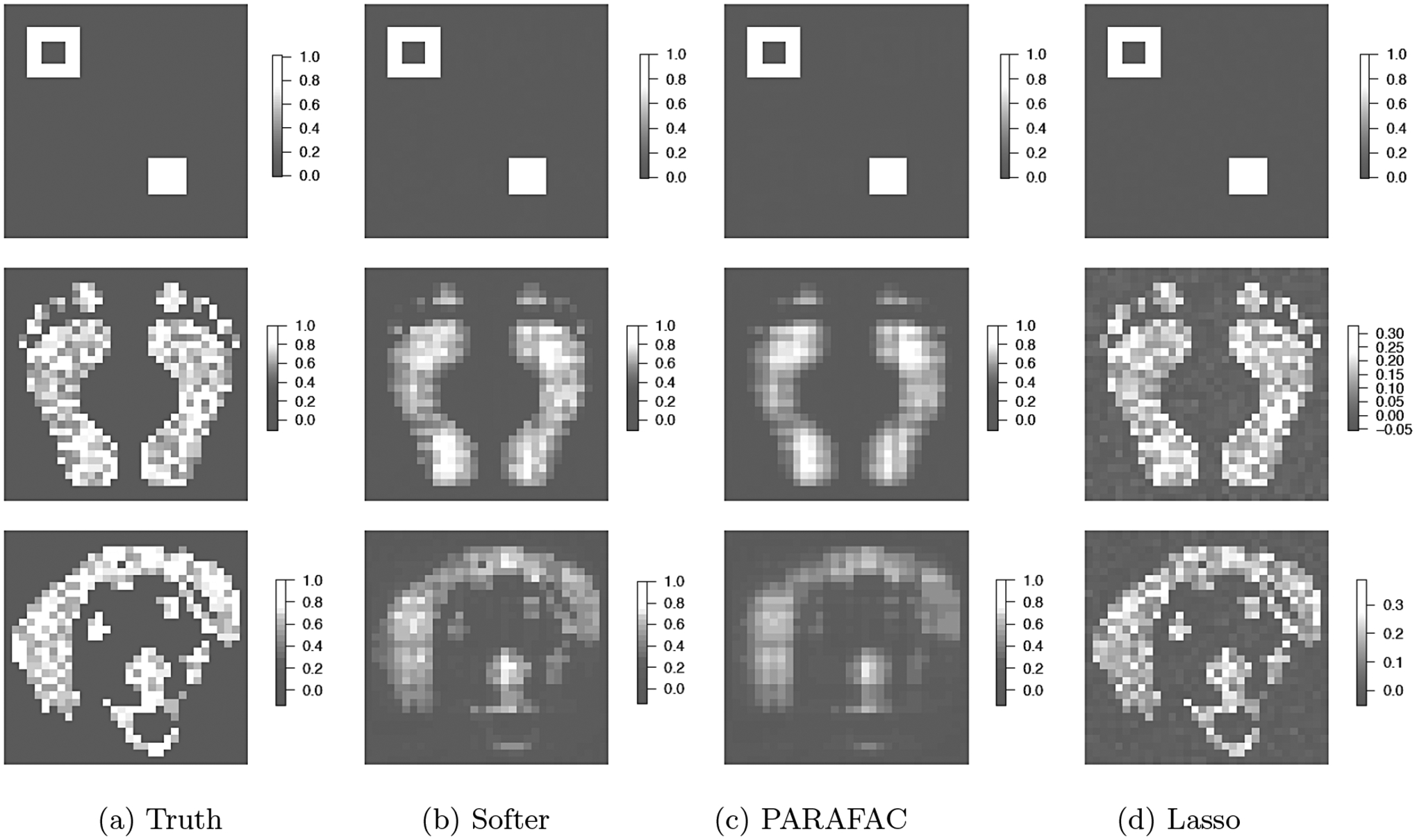
Simulation results for alternative coefficient matrices. True and average estimated coefficient matrix based on Softer, hard PARAFAC, and Lasso. The color scale is the same for the truth, and the Softer and hard PARAFAC approaches, but different for the Lasso because the average estimated coefficients for the Lasso is much smaller.

**Figure D.4:**
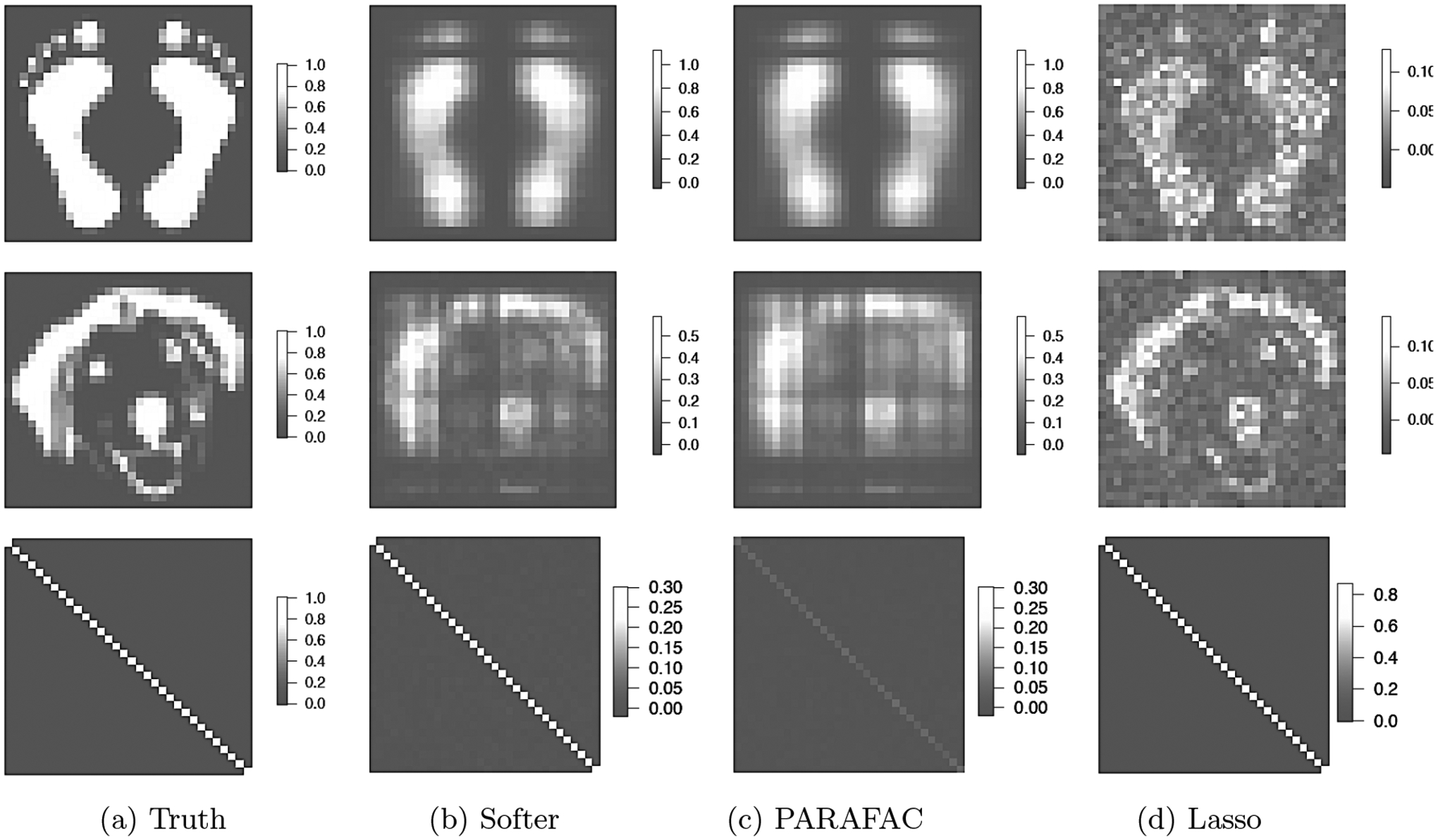
True and average estimated coefficient matrix for Softer, hard PARAFAC, and Lasso. Note that the true matrices might be shown at a different color scale than the estimated ones.

**Figure D.5:**
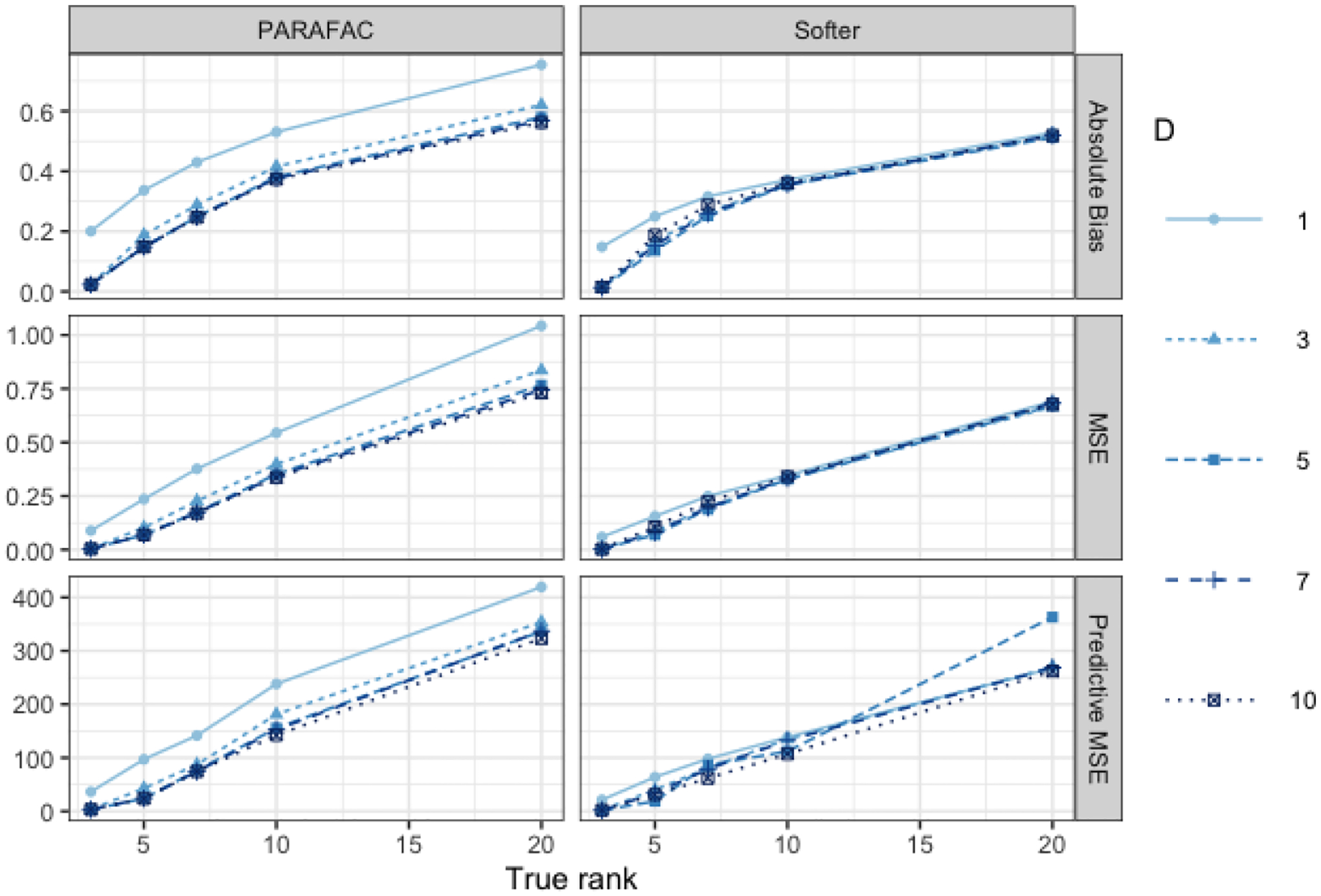
Bias, MSE and predictive MSE (rows) for varying rank of the true coefficient matrix (horizontal axis) for the hard PARAFAC and Softer (columns) with various choices of the algorithmic rank *D*.

**Figure E.1:**
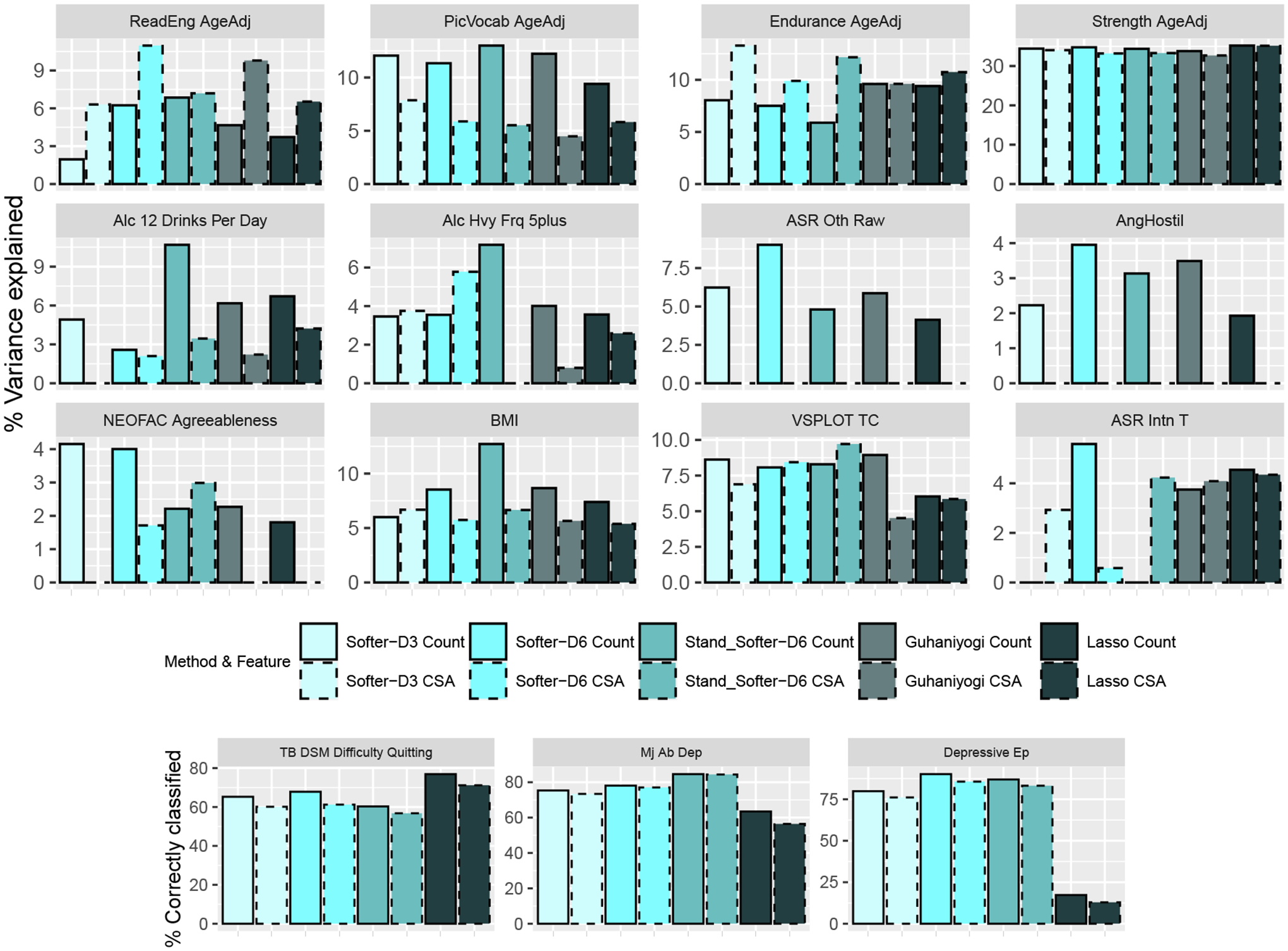
Additional study results showing predicted variable explained and percentage of correctly classified held-out data based on a larger set of approaches.

## Appendix A. Proofs

In this appendix, we provide proofs to all propositions.

### Proof of Proposition 1

#### Expectation.

We use *S*, *Z* and *W* to denote the collection of $\sigma_{k}^{2}$, *ζ*^(*d*)^ and $w_{k,j_{k}}^{\left(d\right)}$, over *k*, *d*, and (*k*, *j*_*k*_, *d*) accordingly. We start by noting that

$$\beta_{k,\underset{˜}{j}}^{\left(d\right)}|\sigma_{k}^{2},\zeta^{\left(d\right)},\tau_{\gamma },w_{k,j_{k}}^{\left(d\right)}\sim N\left(0,\sigma_{k}^{2}\zeta^{\left(d\right)}+\tau_{\gamma }\zeta^{\left(d\right)}w_{k,j_{k}}^{\left(d\right)}\right),$$

and, if $\left(k,j_{k},d\right)\neq \left(k^{\prime },j_{k}^{\prime },d^{\prime }\right)$

(A.1)
$$\beta_{k,\underset{˜}{j}}^{\left(d\right)}⫫\beta_{k^{\prime },{\underset{˜}{j}}^{\prime }}^{\left(d^{\prime }\right)}|S,Z,W,\tau_{\gamma }.$$

Note that $\beta_{k,\underset{˜}{j}}^{\left(d\right)}$ is *not* independent of $\beta_{k,{\underset{˜}{j}}^{\prime }}^{\left(d\right)}$ conditional on (*S*, *Z*, *W*, *τ*_*γ*_) when $j_{k}=j_{k}^{\prime }$ due to their shared dependence on $\gamma_{k,j_{k}}^{\left(d\right)}$. Then,

$$E\left(B_{\underset{˜}{j}}|S,Z,W,\tau_{\gamma }\right)=E\left(\overset{D}{\underset{d=1}{\sum }}\overset{K}{\underset{k=1}{\prod }}\beta_{k,\underset{˜}{j}}^{\left(d\right)}|S,Z,W,\tau_{\gamma }\right)=\overset{D}{\underset{d=1}{\sum }}\overset{K}{\underset{k=1}{\prod }}E\left(\beta_{k,\underset{˜}{j}}^{\left(d\right)}|S,Z,W,\tau_{\gamma }\right)=0$$

So, a priori, all elements of the coefficient tensor have mean 0, $E\left(B_{\underset{˜}{j}}\right)=0$.

#### Variance.

Furthermore, we have

$$\text{Var}\left(B_{\underset{˜}{j}}\right)=E\left\{\text{Var}\left(B_{\underset{˜}{j}}|S,Z,W,\tau_{\gamma }\right)\right\}=E\left\{\text{Var}\left(\overset{D}{\underset{d=1}{\sum }}\overset{K}{\underset{k=1}{\prod }}\beta_{k,\underset{˜}{j}}^{\left(d\right)}|S,Z,W,\tau_{\gamma }\right)\right\}.$$

Since the $\beta_{k,\underset{˜}{j}}^{\left(d\right)}$ are conditionally independent across *d*, $\prod_{k=1}^{K}\beta_{k,\underset{˜}{j}}^{\left(d\right)}$ are also conditionally independent across *d*. Moreover, the terms of the product $\beta_{k,\underset{˜}{j}}^{\left(d\right)}$ are independent across *k* and are mean-zero random variables, implying that $\prod_{k=1}^{K}\beta_{k,\underset{˜}{j}}^{\left(d\right)}$ are mean zero variables. Note here that two independent mean-zero random variables *A*, *B* satisfy that Var(*AB*) = Var(*A*)Var(*B*). Then,

$$\text{Var}\left(B_{\underset{˜}{j}}\right)=E\left\{\overset{D}{\underset{d=1}{\sum }}\overset{K}{\underset{k=1}{\prod }}\text{ Var}\left(\beta_{k,\underset{˜}{j}}^{\left(d\right)}|S,Z,W,\tau_{\gamma }\right)\right\}\;=E\left\{\overset{D}{\underset{d=1}{\sum }}\overset{K}{\underset{k=1}{\prod }}\zeta^{\left(d\right)}\left(\sigma_{k}^{2}+\tau_{\gamma }w_{k,j_{k}}^{\left(d\right)}\right)\right\}\;=E_{Z}\left\{\overset{D}{\underset{d=1}{\sum }}{\left(\zeta^{\left(d\right)}\right)}^{K}\right\}E_{S,W,\tau_{\gamma }}\left\{\overset{K}{\underset{k=1}{\prod }}\left(\sigma_{k}^{2}+\tau_{\gamma }w_{k,j_{k}}^{\left(d\right)}\right)\right\},$$

where in the last equation we used that, a priori, *Z* ⫫ (*S*, *W*, *τ*_*γ*_) to write $E_{S,W,\tau_{\gamma }|Z}$ as $E_{S,W,\tau_{\gamma }}$, and separate the two expectations.

However, $\sigma_{k}^{2}+\tau_{\gamma }w_{k,j_{k}}^{\left(d\right)}$ are not independent of each other for different values of *k* since they all involve the same parameter *τ*_*γ*_. We overcome this difficulty in calculating the expectation of the product by writing $\prod_{k=1}^{K}\left(\sigma_{k}^{2}+\tau_{\gamma }w_{k,j_{k}}^{\left(d\right)}\right)=\sum_{l=0}^{K}c_{l}\tau_{\gamma }^{l}$, where

$$c_{l}=\underset{K\subset \left\{1,2,\ldots ,K\right\}:\left|K\right|=l}{\sum }\left(\underset{k\in K}{\prod }w_{k,j_{k}}^{\left(d\right)}\underset{k\notin K}{\prod }\sigma_{k}^{2}\right).$$

So, for every power of *τ*_*γ*_, $\tau_{\gamma }^{l}$, *l* ∈ {0, 1, …, *K*}, the corresponding coefficient is a sum of all terms involving *l* distinct *w*’s and *K* − *l* distinct *σ*^2^’s. For example, for *K* = 2, $c_{1}=w_{1,j_{1}}^{\left(d\right)}\sigma_{2}^{2}+\sigma_{1}^{2}w_{2,j_{2}}^{\left(d\right)}$. Writing the product in this way, separates the terms ($w_{k,j_{k}}^{\left(d\right)}$, $\sigma_{k}^{2}$) from *τ*_*γ*_, which are a priori independent. Then,

$$\text{Var}\left(B_{\underset{˜}{j}}\right)=E_{Z}\left\{\overset{D}{\underset{d=1}{\sum }}{\left(\zeta^{\left(d\right)}\right)}^{K}\right\}E_{S,W,\tau_{\gamma }}\left(\overset{K}{\underset{l=0}{\sum }}c_{l}\tau_{\gamma }^{l}\right)=E_{Z}\left\{\overset{D}{\underset{d=1}{\sum }}{\left(\zeta^{\left(d\right)}\right)}^{K}\right\}\left\{\overset{K}{\underset{l=0}{\sum }}E\left(\tau_{\gamma }^{l}\right)E_{S,W}\left(c_{l}\right)\right\}.$$

We continue by studying $E_{S,W}\left(c_{l}\right)=\sum_{K:\left|K\right|=l}E_{S,W}\left(\prod_{k\in K}w_{k,j_{k}}^{\left(d\right)}\prod_{k\notin K}\sigma_{k}^{2}\right)$. Note that since all parameters $\left\{w_{k,j_{k}}^{\left(d\right)},\sigma_{k}^{2}\right\}k$ for fixed *j*_*k*_ are a priori independent (any dependence in the $w_{k,j_{k}}^{\left(d\right)}$ exists across *j*_*k*_ of the same mode due to the common value $\lambda_{k}^{\left(d\right)}$), $E_{S,W}\left(c_{l}\right)=\sum_{K:\left|K\right|=l}\left(\prod_{k\in K}E_{W}\left(w_{k,j_{k}}^{\left(d\right)}\right)\prod_{k\notin K}E_{S}\left(\sigma_{k}^{2}\right)\right)$. Now, note that $E\left(\sigma_{k}^{2}\right)=\frac{a_{\sigma }}{b_{\sigma }}$, and

$$E_{W}\left(w_{k,j_{k}}^{\left(d\right)}\right)=E_{\Lambda }\left\{E_{W|\Lambda }\left[w_{k,j_{k}}^{\left(d\right)}\right]\right\}=2E_{\Lambda }\left\{{\left(\lambda_{k}^{\left(d\right)}\right)}^{-2}\right\}.$$

Since $\lambda_{k}^{\left(d\right)}\sim \Gamma \left(a_{\lambda },b_{\lambda }\right),1/\lambda_{k}^{\left(d\right)}\sim IG\left(a_{\lambda },b_{\lambda }\right)$, we have that

$$E\left\{{\left(1/\lambda_{k}^{\left(d\right)}\right)}^{2}\right\}=\text{Var}\left(1/\lambda_{k}^{\left(d\right)}\right)+E^{2}\left(1/\lambda_{k}^{\left(d\right)}\right)=\frac{b_{\lambda }^{2}}{\left(a_{\lambda }-1\right)\left(a_{\lambda }-2\right)},a_{\lambda }>2.$$

Putting this together, we have that, for *a*_*λ*_ > 2,

$$E_{S,W}\left(c_{l}\right)=\left(\begin{array}{c} K\\ l \end{array}\right){\left\{\frac{2b_{\lambda }^{2}}{\left(a_{\lambda }-1\right)\left(a_{\lambda }-2\right)}\right\}}^{l}{\left(\frac{a_{\sigma }}{b_{\sigma }}\right)}^{K-l}.$$

Further, since *τ*_*γ*_ ~ Γ(*a*_*τ*_, *b*_*τ*_), we have that

$$E\left(\tau_{\gamma }^{l}\right)=\frac{b_{\tau }^{a_{\tau }}}{\Gamma \left(a_{\tau }\right)}\int \tau_{\gamma }^{a_{\tau }+l-1}\text{exp}\left\{-b_{\tau }\tau_{\gamma }\right\}\text{ d}\tau_{\gamma }=\frac{\Gamma \left(a_{\tau }+l\right)}{\Gamma \left(a_{\tau }\right)b_{\tau }^{l}}=\frac{\rho_{l}}{b_{\tau }^{l}},$$

for *ρ*_*l*_ = 1 if *l* = 0, and *ρ*_*l*_ = *a*_*τ*_(*a*_*τ*_ + 1) … (*a*_*τ*_ + *l* − 1) if *l* ≥ 1. Lastly, since ***ζ*** ~ *Dir*(*α*/*D*, *α*/*D*, …, *α*/*D*), we have that *ζ*^(*d*)^ ~ *Beta*(*α*/*D*, (*D* − 1)*α*/*D*), and

$$E\left\{{\left(\zeta^{\left(d\right)}\right)}^{K}\right\}=\overset{K-1}{\underset{r=0}{\prod }}\frac{\alpha /D+r}{\alpha +r}$$

Combining all of these, we can write the prior variance for entries $B_{\underset{˜}{j}}$ of the coefficient tensor as

$$\text{Var}\left(B_{\underline{j}}\right)=E_{Z}\left[\overset{D}{\underset{d=1}{\sum }}{\left(\zeta^{\left(d\right)}\right)}^{K}\right]\overset{K}{\underset{l=0}{\sum }}\rho_{l}\left(\begin{array}{c} K\\ l \end{array}\right){\left\{\frac{2b_{\lambda }^{2}}{b_{\tau }\left(a_{\lambda }-1\right)\left(a_{\lambda }-2\right)}\right\}}^{l}{\left(\frac{a_{\sigma }}{b_{\sigma }}\right)}^{K-l}\;=\left\{D\overset{K-1}{\underset{r=0}{\prod }}\frac{\alpha /D+r}{\alpha +r}\right\}\left[\overset{K}{\underset{l=0}{\sum }}\rho_{l}\left(\begin{array}{c} K\\ l \end{array}\right){\left\{\frac{2b_{\lambda }^{2}}{b_{\tau }\left(a_{\lambda }-1\right)\left(a_{\lambda }-2\right)}\right\}}^{l}{\left(\frac{a_{\sigma }}{b_{\sigma }}\right)}^{K-l}\right].$$

#### Covariance.

Since $E\left(B_{\underset{˜}{j}}|S,Z,W,\tau_{\gamma }\right)=0$, we have that

$$\text{Cov}\left(B_{\underset{˜}{j}},B_{{\underset{˜}{j}}^{\prime }}\right)=E\left\{\text{Cov}\left(B_{\underset{˜}{j}},B_{{\underset{˜}{j}}^{\prime }}|S,Z,W,\tau_{\gamma }\right)\right\}.$$

Remember from Equation A.1 that, when at least one of *k*, *j*_*k*_, *d* are different, $\beta_{k,\underset{˜}{j}}^{\left(d\right)}⫫\beta_{k^{\prime },{\underset{˜}{j}}^{\prime }}^{\left(d^{\prime }\right)}|S,Z,W,\tau_{\gamma }$. However, that is not true when $\left(k,j_{k},d\right)=\left(k^{\prime },j_{k}^{\prime },d^{\prime }\right)$, even if $\underset{˜}{j}\neq \underset{˜}{j}$. We write

$$E\left\{\text{Cov}\left(B_{\underset{˜}{j}},B_{{\underset{˜}{j}}^{\prime }}|S,Z,W,\tau_{\gamma }\right)\right\}=E\left\{\text{Cov}\left(\sum_{d=1}^{D}\prod_{k=1}^{K}\beta_{k,\underset{˜}{j}}^{\left(d\right)},\sum_{d=1}^{D}\prod_{k=1}^{K}\beta_{k,{\underset{˜}{j}}^{\prime }}^{\left(d\right)}|S,Z,W,\tau_{\gamma }\right)\right\}\;=\sum_{d,d^{\prime }=1}^{D}E\left\{\text{Cov}\left(\prod_{k=1}^{K}\beta_{k,\underset{˜}{j}}^{\left(d\right)},\prod_{k=1}^{K}\beta_{k,{\underset{˜}{j}}^{\prime }}^{\left(d^{\prime }\right)}|S,Z,W,\tau_{\gamma }\right)\right\}.$$

However,

$$\text{Cov}\left(\overset{K}{\underset{k=1}{\prod }}\beta_{k,\underset{˜}{j}}^{\left(d\right)},\overset{K}{\underset{k=1}{\prod }}\beta_{k,{\underset{˜}{j}}^{\prime }}^{\left(d^{\prime }\right)}|S,Z,W,\tau_{\gamma }\right)=E\left(\overset{K}{\underset{k=1}{\prod }}\beta_{k,\underset{˜}{j}}^{\left(d\right)}\beta_{k,{\underset{˜}{j}}^{\prime }}^{\left(d^{\prime }\right)}|S,Z,W,\tau_{\gamma }\right)-E\left(\overset{K}{\underset{k=1}{\prod }}\beta_{k,\underset{˜}{j}}^{\left(d\right)}|S,Z,W,\tau_{\gamma }\right)E\left(\overset{K}{\underset{k=1}{\prod }}\beta_{k,{\underset{˜}{j}}^{\prime }}^{\left(d^{\prime }\right)}|S,Z,W,\tau_{\gamma }\right)=\;E\left(\overset{K}{\underset{k=1}{\prod }}\beta_{k,\underset{˜}{j}}^{\left(d\right)}\beta_{k,{\underset{˜}{j}}^{\prime }}^{\left(d^{\prime }\right)}|S,Z,W,\tau_{\gamma }\right),$$

where the last equation holds because the $\beta_{k,\underset{˜}{j}}^{\left(d\right)}$ are independent of each other across *k* conditional on *S*, *Z*, *W*, *τ*_*γ*_ and have mean zero. Furthermore, since the $\beta_{k,\underset{˜}{j}}^{\left(d\right)}$ are conditionally independent across *d*, we have that for *d* ≠ *d*′, $\text{Cov}\left(\prod_{k=1}^{K}\beta_{k,\underset{˜}{j}}^{\left(d\right)},\prod_{k=1}^{K}\beta_{k,{\underset{˜}{j}}^{\prime }}^{\left(d^{\prime }\right)}|S,Z,W,\tau_{\gamma }\right)=0$. So we only need to study the conditional covariance for *d* = *d*′. For Γ representing the set of all $\gamma_{k,j_{k}}^{\left(d\right)}$, we write

$$E\left(\overset{K}{\underset{k=1}{\prod }}\beta_{k,\underset{˜}{j}}^{\left(d\right)}\beta_{k,{\underset{˜}{j}}^{\prime }}^{\left(d\right)}|S,Z,W,\tau_{\gamma }\right)=E\left\{E\left(\overset{K}{\underset{k=1}{\prod }}\beta_{k,\underset{˜}{j}}^{\left(d\right)}\beta_{k,{\underset{˜}{j}}^{\prime }}^{\left(d\right)}|\Gamma ,S,Z,W,\tau_{\gamma }\right)|S,Z,W,\tau_{\gamma }\right\}.$$

Conditional on Γ, *S*, *Z*, *W*, *τ*_*γ*_, and as long as $\underset{˜}{j}\neq {\underset{˜}{j}}^{\prime }$, the $\beta_{k,\underset{˜}{j}}^{\left(d\right)}$ are independent across all indices, even if they have all of *k*, *j*_*k*_, *d* common, leading to

$$E\left(\prod_{k=1}^{K}\beta_{k,\underset{˜}{j}}^{\left(d\right)}\beta_{k,{\underset{˜}{j}}^{\prime }}^{\left(d\right)}|S,Z,W,\tau_{\gamma }\right)=\;=E\left\{\prod_{k=1}^{K}E\left(\beta_{k,\underset{˜}{j}}^{\left(d\right)}|\Gamma ,S,Z,W,\tau_{\gamma }\right)E\left(\beta_{k,{\underset{˜}{j}}^{\prime }}^{\left(d\right)}|\Gamma ,S,Z,W,\tau_{\gamma }\right)|S,Z,W,\tau_{\gamma }\right\}\;=E\left(\prod_{k=1}^{K}\gamma_{k,j_{k}}^{\left(d\right)}\gamma_{k,j_{k}^{\prime }}^{\left(d\right)}|S,Z,W,\tau_{\gamma }\right)\;=\prod_{k=1}^{K}E\left(\gamma_{k,j_{k}}^{\left(d\right)}\gamma_{k,j_{k}^{\prime }}^{\left(d\right)}|S,Z,W,\tau_{\gamma }\right)\;=\prod_{k:j_{k}=j_{k}^{\prime }}E\left({\left(\gamma_{k,j_{k}}^{\left(d\right)}\right)}^{2}|S,Z,W,\tau_{\gamma }\right)\times \prod_{k:j_{k}\neq j_{k}^{\prime }}E\left(\gamma_{k,j_{k}}^{\left(d\right)}|S,Z,W,\tau_{\gamma }\right)E\left(\gamma_{k,j_{k}^{\prime }}^{\left(d\right)}|S,Z,W,\tau_{\gamma }\right)\;=0$$

where the first equality holds because $\underset{˜}{j}\neq {\underset{˜}{j}}^{\prime }$, the third equality holds because the $\gamma_{k,j_{k}}^{\left(d\right)}$ are conditionally independent across *k*, and the fourth equality holds because they are conditionally independent across *j*_*k*_.

### Proof of Proposition 2

We want Var(*B*_*j*_) = *V** and *AV* = *AV**. The second target will be achieved when $\text{Var}\left(B_{\underset{˜}{j}}\right)/{\text{Var}}^{\text{hard}}\left(B_{\underset{˜}{j}}\right)={\left(1-AV^{*}\right)}^{-1}$. Since $\frac{a_{\sigma }}{b_{\sigma }}$ is the quantity driving the soft PARAFAC’s additional variability we use this condition to acquire a form for $\frac{a_{\sigma }}{b_{\sigma }}$ as a function of the remaining hyperparameters.

$$\frac{\text{Var}\left(B_{\underset{˜}{j}}\right)}{{\text{Var}}^{\text{hard}}\left(B_{\underset{˜}{j}}\right)}=\frac{\sum_{l=0}^{2}\frac{\rho_{l}}{b_{\tau }^{l}}\left(\begin{array}{l} 2\\ l \end{array}\right){\left\{\frac{2b_{\lambda }^{2}}{\left(a_{\lambda }-1\right)\left(a_{\lambda }-2\right)}\right\}}^{l}{\left(\frac{a_{\sigma }}{b_{\sigma }}\right)}^{2-l}}{\frac{\rho_{2}}{b_{\tau }^{2}}{\left\{\frac{2b_{\lambda }^{2}}{\left(a_{\lambda }-1\right)\left(a_{\lambda }-2\right)}\right\}}^{2}}\;=\sum_{l=0}^{2}\left(\begin{array}{l} 2\\ l \end{array}\right)\frac{\rho_{l}}{\rho_{2}}{\left\{\frac{2b_{\lambda }^{2}}{b_{\tau }\left(a_{\lambda }-1\right)\left(a_{\lambda }-2\right)}\right\}}^{l-2}{\left(\frac{a_{\sigma }}{b_{\sigma }}\right)}^{2-l}\;=\frac{1}{\rho_{2}}{\left\{\frac{2b_{\lambda }^{2}}{b_{\tau }\left(a_{\lambda }-1\right)\left(a_{\lambda }-2\right)}\right\}}^{-2}{\left(\frac{a_{\sigma }}{b_{\sigma }}\right)}^{2}+2\frac{\rho_{1}}{\rho_{2}}{\left\{\frac{2b_{\lambda }^{2}}{b_{\tau }\left(a_{\lambda }-1\right)\left(a_{\lambda }-2\right)}\right\}}^{-1}\frac{a_{\sigma }}{b_{\sigma }}+1\;=\frac{1}{a_{\tau }\left(a_{\tau }+1\right)}{\left\{\frac{2b_{\lambda }^{2}}{b_{\tau }\left(a_{\lambda }-1\right)\left(a_{\lambda }-2\right)}\right\}}^{-2}{\left(\frac{a_{\sigma }}{b_{\sigma }}\right)}^{2}+\frac{2}{a_{\tau }+1}{\left\{\frac{2b_{\lambda }^{2}}{b_{\tau }\left(a_{\lambda }-1\right)\left(a_{\lambda }-2\right)}\right\}}^{-1}\frac{a_{\sigma }}{b_{\sigma }}+1$$

Therefore, in order for $\text{Var}\left(B_{\underset{˜}{j}}\right)/{\text{Var}}^{\text{hard}}\left(B_{\underset{˜}{j}}\right)={\left(1-AV^{*}\right)}^{-1}$, $\frac{a_{\sigma }}{b_{\sigma }}$ is the solution to a second degree polynomial. We calculate the *positive* root of this polynomial.

$$\begin{array}{l} \Delta =\frac{4}{{\left(a_{\tau }+1\right)}^{2}}{\left\{\frac{2b_{\lambda }^{2}}{b_{\tau }\left(a_{\lambda }-1\right)\left(a_{\lambda }-2\right)}\right\}}^{-2}-\frac{4}{a_{\tau }\left(a_{\tau }+1\right)}{\left\{\frac{2b_{\lambda }^{2}}{b_{\tau }\left(a_{\lambda }-1\right)\left(a_{\lambda }-2\right)}\right\}}^{-2}\left(1-{\left(1-AV^{*}\right)}^{-1}\right)\\ =\frac{4}{{\left(a_{\tau }+1\right)}^{2}}{\left\{\frac{2b_{\lambda }^{2}}{b_{\tau }\left(a_{\lambda }-1\right)\left(a_{\lambda }-2\right)}\right\}}^{-2}\left[1-\frac{a_{\tau }+1}{a_{\tau }}\left\{1-{\left(1-AV^{*}\right)}^{-1}\right\}\right]>0. \end{array}$$

Since $\frac{a_{\sigma }}{b_{\sigma }}$ is positive, we have that $\frac{a_{\sigma }}{b_{\sigma }}$ is equal to

(A.2)
$$\frac{-\frac{2}{a_{\tau }+1}{\left\{\frac{2b_{\lambda }^{2}}{b_{\tau }\left(a_{\lambda }-1\right)\left(a_{\lambda }-2\right)}\right\}}^{-1}+\sqrt{\frac{4}{{\left(a_{\tau }+1\right)}^{2}}{\left\{\frac{2b_{\lambda }^{2}}{b_{\tau }\left(a_{\lambda }-1\right)\left(a_{\lambda }-2\right)}\right\}}^{-2}\left[1-\frac{a_{\tau }+1}{a_{\tau }}\left\{1-{\left(1-AV^{*}\right)}^{-1}\right\}\right]}}{\frac{2}{a_{\tau }\left(a_{\tau }+1\right)}{\left\{\frac{2b_{\lambda }^{2}}{b_{\tau }\left(a_{\lambda }-1\right)\left(a_{\lambda }-2\right)}\right\}}^{-2}}\;=\frac{-1+\sqrt{1-\frac{a_{\tau }+1}{a_{\tau }}\left\{1-{\left(1-AV^{*}\right)}^{-1}\right\}}}{\frac{1}{a_{\tau }}{\left\{\frac{2b_{\lambda }^{2}}{b_{\tau }\left(a_{\lambda }-1\right)\left(a_{\lambda }-2\right)}\right\}}^{-1}}\;=\frac{a_{\tau }}{b_{\tau }}\frac{2b_{\lambda }^{2}}{\left(a_{\lambda }-1\right)\left(a_{\lambda }-2\right)}\left\{\sqrt{1-\frac{a_{\tau }+1}{a_{\tau }}\left\{1-{\left(1-AV^{*}\right)}^{-1}\right\}}-1\right\}.$$

Denoting $\xi =1-\frac{a_{\tau }+1}{a_{\tau }}\left\{1-{\left(1-AV^{*}\right)}^{-1}\right\}$ and substituting the form of $\frac{a_{\sigma }}{b_{\sigma }}$ in $\text{Var}\left(B_{\underset{˜}{j}}\right)$ we have that

$$\text{Var}\left(B_{\underset{˜}{j}}\right)=C\sum_{l=0}^{2}\left(\begin{array}{l} 2\\ l \end{array}\right)\frac{\rho_{l}}{b_{\tau }^{l}}{\left\{\frac{2b_{\lambda }^{2}}{\left(a_{\lambda }-1\right)\left(a_{\lambda }-2\right)}\right\}}^{2}{\left(\frac{a_{\tau }}{b_{\tau }}\right)}^{2-l}{\left(\sqrt{\xi }-1\right)}^{2-l}\;=C{\left\{\frac{2b_{\lambda }^{2}}{\left(a_{\lambda }-1\right)\left(a_{\lambda }-2\right)}\right\}}^{2}{\left(\frac{a_{\tau }}{b_{\tau }}\right)}^{2}\sum_{l=0}^{2}\left(\begin{array}{l} 2\\ l \end{array}\right)\frac{\rho_{l}}{a_{\tau }^{l}}{\left(\sqrt{\xi }-1\right)}^{2-l}\;=C{\left\{\frac{2b_{\lambda }^{2}}{\left(a_{\lambda }-1\right)\left(a_{\lambda }-2\right)}\right\}}^{2}{\left(\frac{a_{\tau }}{b_{\tau }}\right)}^{2}\left\{{\left(\sqrt{\xi }-1\right)}^{2}+2(\sqrt{\xi }-1)+1+\frac{1}{a_{\tau }}\right\}\;=C{\left\{\frac{2b_{\lambda }^{2}}{\left(a_{\lambda }-1\right)\left(a_{\lambda }-2\right)}\right\}}^{2}{\left(\frac{a_{\tau }}{b_{\tau }}\right)}^{2}\left\{\xi +\frac{1}{a_{\tau }}\right\}$$

Also,

$$\xi +\frac{1}{a_{\tau }}=1-\frac{a_{\tau }+1}{a_{\tau }}\left\{1-{\left(1-AV^{*}\right)}^{-1}\right\}+\frac{1}{a_{\tau }}=\frac{a_{\tau }+1}{a_{\tau }}{\left(1-AV^{*}\right)}^{-1}$$

leading to

(A.3)
$$\text{Var}\left(B_{\underset{˜}{j}}\right)=V^{*}\Leftrightarrow \frac{2b_{\lambda }^{2}}{\left(a_{\lambda }-1\right)\left(a_{\lambda }-2\right)}=\frac{b_{\tau }}{a_{\tau }}\sqrt{\frac{V^{*}\left(1-AV^{*}\right)a_{\tau }}{C\left(a_{\tau }+1\right)}}.$$

Substituting Equation A.3 back into Equation A.2, we have that

$$\frac{a_{\sigma }}{b_{\sigma }}=\sqrt{\frac{V^{*}\left(1-AV^{*}\right)a_{\tau }}{C\left(a_{\tau }+1\right)}}\left\{\sqrt{1-\frac{a_{\tau }+1}{a_{\tau }}\left\{1-{\left(1-AV^{*}\right)}^{-1}\right\}}-1\right\}.$$

### Proof of Proposition 3

Start by noting that

$$\pi_{B}\left(B_{\epsilon }^{\infty }\left(B^{0}\right)\right)=E_{\Gamma ,S,Z}\left[p\left(B:\underset{\underset{˜}{j}}{\text{max}}\left|B_{\underset{˜}{j}}^{0}-B_{\underset{˜}{j}}\right|<\epsilon |\Gamma ,S,Z\right)\right],$$

where Γ, *S*, *Z* are as defined in the proof of Proposition 1. Then, take $\epsilon^{*}=\sqrt[{K}]{\epsilon /(2(D-1))}$ and write

$$\begin{array}{l} p\left(B:\underset{\underset{˜}{j}}{\text{max}}\left|B_{\underset{˜}{j}}^{0}-B_{\underset{˜}{j}}\right|<\epsilon |\Gamma ,S,Z\right)\\ \geq p\left(B:\underset{\underset{˜}{j}}{\text{max}}\left|B_{\underset{˜}{j}}^{0}-B_{\underset{˜}{j}}\right|<\epsilon |\Gamma ,S,Z\left\{\left|\beta_{k,\underset{˜}{j}}^{\left(d\right)}\right|<\epsilon^{*}\text{, all }k,\underset{˜}{j},\text{ and }d\geq 2\right\}\right)\times p\left(\left|\beta_{k,\underset{˜}{j}}^{\left(d\right)}\right|<\epsilon^{*},\text{ all }k,\underset{˜}{j},\text{ and }d\geq 2\}|\Gamma ,S,Z\right). \end{array}$$

Conditional on Γ, *S*, *Z*, $\beta_{k,\underset{˜}{j}}^{\left(d\right)}$ are independent normal variables with positive weight in an *ϵ**-neighborhood of 0, implying that $p\left(\left|\beta_{k,\underset{˜}{j}}^{\left(d\right)}\right|<\epsilon^{*},\text{ all }k,\underset{˜}{j},\text{ and }d\geq 2\}|\Gamma ,S,Z\right)>0$.

Remember from Equation 5 that $B=\sum_{d=1}^{D}B_{1}^{\left(d\right)}\circ B_{2}^{\left(d\right)}\circ \ldots \circ B_{K}^{\left(d\right)}$, and denote $B^{\left(d\right)}=B_{1}^{\left(d\right)}\circ B_{2}^{\left(d\right)}\circ \ldots \circ B_{K}^{\left(d\right)}$. Then, $B_{\underset{˜}{j}}=B_{\underset{˜}{j}}^{\left(1\right)}+B_{\underset{˜}{j}}^{\left(2\right)}+\ldots +B_{\underset{˜}{j}}^{\left(D\right)}$. Note that

$$p\left(B:\underset{\underset{˜}{j}}{\text{max}}\left|B_{\underset{˜}{j}}^{0}-B_{\underset{˜}{j}}\right|<\epsilon |\Gamma ,S,Z\left\{\left|\beta_{k,\underset{˜}{j}}^{\left(d\right)}\right|<\epsilon^{*},\text{ all }k,\underset{˜}{j},\text{ and }d\geq 2\right\}\right)\;\geq p\left(B:\underset{\underset{˜}{j}}{\text{max}}\left|B_{\underset{˜}{j}}^{0}-B_{\underset{˜}{j}}^{\left(1\right)}\right|<\epsilon /2|\Gamma ,S,Z\left\{\left|\beta_{k,\underset{˜}{j}}^{\left(d\right)}\right|<\epsilon^{*},\text{ all }k,\underset{˜}{j},\text{ and }d\geq 2\right\}\right)\;=p\left(B:\underset{\underset{˜}{j}}{\text{max}}\left|B_{\underset{˜}{j}}^{0}-B_{\underset{˜}{j}}^{\left(1\right)}\right|<\epsilon /2|\Gamma ,S,Z\right),$$

where the equality holds because the entries of ***B***^(1)^ are independent of all $\beta_{k,\underset{˜}{j}}^{\left(d\right)}$ for *d* ≥ 2 conditional on Γ, *S*, *Z*, and the inequality holds because $\left|B_{\underset{˜}{j}}^{0}-B_{\underset{˜}{j}}^{\left(1\right)}\right|<\epsilon /2$ and $\left|\beta_{k,\underset{˜}{j}}^{\left(d\right)}\right|<\epsilon^{*}$ for *d* ≥ 2 implies that

$$\left|B_{\underset{˜}{j}}^{0}-B_{\underset{˜}{j}}\right|=\left|B_{\underset{˜}{j}}^{0}-B_{\underset{˜}{j}}^{\left(1\right)}-B_{\underset{˜}{j}}^{\left(2\right)}-\cdots -B_{\underset{˜}{j}}^{\left(D\right)}\right|\;\leq \left|B_{\underset{˜}{j}}^{0}-B_{\underset{˜}{j}}^{\left(1\right)}\right|+\left|B_{\underset{˜}{j}}^{\left(2\right)}\right|+\cdots +\left|B_{\underset{˜}{j}}^{\left(D\right)}\right|\;<\epsilon /2+(D-1){\left(\epsilon^{*}\right)}^{K}=\epsilon .$$

Since all of $\beta_{k,\underset{˜}{j}}^{\left(d\right)}$ are independent conditional on Γ, *S*, *Z*, we have that

$$p\left(B:\underset{\underset{˜}{j}}{\text{max}}\left|B_{\underset{˜}{j}}^{0}-B_{\underset{˜}{j}}^{\left(1\right)}\right|<\epsilon /2|\Gamma ,S,Z\right)=\underset{\underset{˜}{j}}{\prod }p\left(\left|B_{\underset{˜}{j}}^{0}-B_{\underset{˜}{j}}^{\left(1\right)}\right|<\epsilon /2|\Gamma ,S,Z\right)>0,$$

since all entries $B_{\underset{˜}{j}}^{\left(1\right)}$ are products of draws from *K* normal distributions and therefore assign positive weight in all ℝ, including the *ϵ*/2–neighborhood of $B_{\underset{˜}{j}}^{0}$.

Putting all of this together, we have the desired result that $\pi_{B}\left(B_{\epsilon }^{\infty }\left(B^{0}\right)\right)>0$.

### Proof of Proposition 4

We show that for any *ϵ* > 0, there exists *ϵ** > 0 such that $\left\{B:{\text{max}}_{\underset{˜}{j}}\left|B_{\underset{˜}{j}}^{0}-B_{\underset{˜}{j}}\right|<\epsilon^{*}\right\}\subseteq \left\{B:KL\left(B^{0},B\right)<\epsilon \right\}$, where

$$KL\left(B_{0},B\right)=\int \text{log}\frac{\phi \left(y;B^{0}\right)}{\phi (y;B)}\phi \left(y;B^{0}\right)\text{d}y,$$

and *ϕ*(*y*; ***B***) is the density of a normal distribution with coefficient tensor ***B*** and variance 1. If we show this, consistency follows from Schwartz (1965).

Assume that there exists *M* such that $\left|X_{\underset{˜}{j}}\right|<M$ for all $\underset{˜}{j}$ with probability 1. For two normal distributions with mean 〈***X***, ***B***^0^〉_*F*_ and 〈***X***, ***B***〉_*F*_ respectively, we have that

$$KL\left(B^{0},B\right)=\frac{1}{2}{\left({\langle X,B^{0}\rangle }_{F}-{\langle X,B^{0}\rangle }_{F}\right)}^{2}\;=\frac{1}{2}{\left[\sum_{\underset{˜}{j}}\left(B_{\underset{˜}{j}}^{0}-B_{\underset{˜}{j}}\right)X_{\underset{˜}{j}}\right]}^{2}\;\leq \frac{1}{2}\left[\sum_{\underset{˜}{j}}{\left(B_{\underset{˜}{j}}^{0}-B_{\underset{˜}{j}}\right)}^{2}\right]\left[\sum_{\underset{˜}{j}}X_{\underset{˜}{j}}^{2}\right]$$

Take $\epsilon^{*}=\sqrt{2\epsilon }/\left(M{\left(p_{1}p_{2}\ldots p_{K}\right)}^{2}\right)$ and consider $B\in B_{\epsilon^{*}}^{\infty }\left(B^{0}\right)$. We will show that ***B*** satisfies that that *KL*(***B***^0^, ***B***) < *ϵ* completing the proof. Note first that

$$\underset{\underset{˜}{j}}{\sum }{\left(B_{\underset{˜}{j}}^{0}-B_{\underset{˜}{j}}\right)}^{2}\leq p_{1}p_{2}\ldots p_{k}\underset{\underset{˜}{j}}{\text{max}}{\left(B_{\underset{˜}{j}}^{0}-B_{\underset{˜}{j}}\right)}^{2}<p_{1}p_{2}\ldots p_{k}{\left(\epsilon^{*}\right)}^{2}=\frac{2\epsilon }{Mp_{1}p_{2}\ldots p_{K}},$$

and since $\left|X_{\underset{˜}{j}}\right|\leq M$ we have that $\sum_{\underset{˜}{j}}X_{\underset{˜}{j}}^{2}\leq Mp_{1}p_{2}\ldots p_{K}$. Putting these results together

$$KL\left(B^{0},B\right)<\frac{1}{2}\frac{2\epsilon }{Mp_{1}p_{2}\ldots p_{K}}Mp_{1}p_{2}\ldots p_{K}=\epsilon .$$

## Appendix B. Hard PARAFAC Error in Estimating the True Matrix

Due to the block structure and subsequent “inflexibility” of the hard PARAFAC approximation, a large number of components *D* might be required in order to adequately approximate a coefficient tensor ***B***. To further demonstrate this, we considered a coefficient matrix ***B*** whose entries ***B***_*ij*_ are centered around (but are not equal to) the entries of a rank-1 matrix of the form *β*_1_ ⊗ *β*_2_. Therefore, even though the matrix has a somewhat rectangular structure, it is not exactly in that form. Using the singular value decomposition (which is the PARAFAC analog for matrices), we considered the quality of the approximation based on *D* factors, for various values of *D*. Figure B.1 shows histograms of the difference of the true entries in ***B*** from the approximated ones. Even for *D* = 20, substantial error remains in estimating the matrix ***B***.

## Appendix C. Alternative Sampling from the Posterior Distribution

The full set of parameters is $\theta =\left\{\mu ,\delta ,\tau^{2},\beta_{k,\underset{˜}{j}}^{\left(d\right)},\gamma_{k,j_{k}}^{\left(d\right)},\sigma_{k}^{2},\zeta^{\left(d\right)},w_{k,j_{k}}^{\left(d\right)},\lambda_{k}^{\left(d\right)},\tau_{\gamma }^{2},\text{ for all }d,k,j_{k},\underset{˜}{j}\right\}$. We use the notation |· and |·, −*y* to denote conditioning on the data and all parameters, and the data and all parameters but *y*, accordingly. Then, our MCMC updates are:

- $(\mu ,\delta )|\cdot \sim N\left(\mu^{*},\Sigma^{*}\right)$, for $\Sigma^{*}={\left(\Sigma_{0}^{-1}+{\tilde{C}}^{T}\tilde{C}/\tau^{2}\right)}^{-1}$, and $\mu^{*}=\Sigma^{*}{\tilde{C}}^{T}R_{B}/\tau^{2}$, where $\tilde{C}$ is the *N* × (*p*+1) matrix with *i*^*th*^ row equal to (1, ***C***_*i*_), and ***R***_*B*_ = (*Y*_1_ – 〈***X***_1_, ***B***〉_*F*_, …, *Y*_*N*_ − 〈***X***_*N*_, ***B***〉_*F*_)^*T*^ is the vector of residuals of the outcome on the tensor predictor.
- $\tau^{2}|\cdot \sim IG\left(a_{\tau }+N/2,b_{\tau }+\sum_{i=1}^{N}\left(Y_{i}-\mu -C_{i}^{T}\delta -{\langle X_{i},B\rangle }_{F}\right)\right)$.
- $\sigma_{k}^{2}|\cdot \sim giG\left(p^{*},a^{*},b^{*}\right)$, for $p^{*}=a_{\sigma }-D\prod_{k=1}^{K}p_{k}/2$, *a** = 2*b*_*σ*_, and $b^{*}=\sum_{d,\underset{˜}{j}}{\left(\beta_{k,\underset{˜}{j}}^{\left(d\right)}-\gamma_{k,j_{k}}^{\left(d\right)}\right)}^{2}/\zeta^{\left(d\right)}$. As a reminder, *X* ~ *giG*(*p*, *a*, *b*) if *p*(*x*) ∝ *x*^*p*−1^ exp{−(*ax* + *b/x*)*/*2}.
- $\gamma_{k,j_{k}}^{\left(d\right)}|\cdot \sim N\left(\mu^{*},\sigma^{2*}\right)$, for $\sigma^{2*}={\left\{{\left(\tau_{\gamma }w_{k,j_{k}}^{\left(d\right)}\zeta^{\left(d\right)}\right)}^{-1}+\left(\underset{l\neq k}{\sum }p_{l}\right)/\left(\sigma_{k}^{2}\zeta^{\left(d\right)}\right)\right\}}^{-1}$, and $\mu^{*}=\sigma^{2*}\left\{\underset{\begin{array}{c} \underset{˜}{j}:{\underset{˜}{j}}_{k}=j_{k} \end{array}}{\sum }\beta_{k,\underset{˜}{j}}^{\left(d\right)}/\left(\sigma_{k}^{2}\zeta^{\left(d\right)}\right)\right\}$.
- $\tau_{\gamma }|\cdot \sim giG\left(a_{\tau }-D\sum_{k}p_{k}/2,2b_{\tau },\sum_{d,k,j_{k}}{\left(\gamma_{k,j_{k}}^{\left(d\right)}\right)}^{2}/\left(\zeta^{\left(d\right)}w_{k,j_{k}}^{\left(d\right)}\right)\right)$
- $w_{k,j_{k}}^{\left(d\right)}|\cdot \sim giG\left(1/2,\lambda_{k}^{2},{\left(\gamma_{k,j_{k}}^{\left(d\right)}\right)}^{2}/\left(\tau_{\gamma }\zeta^{\left(d\right)}\right)\right)$.
- $\left[\lambda_{k}^{\left(d\right)}|\cdot ,-w_{k,j_{k}}^{\left(d\right)}\text{, all }j_{k}\right]\sim \Gamma \left(a_{\lambda }+p_{k},b_{\lambda }+\sum_{j_{k}}\left|\gamma_{k,j_{k}}^{\left(d\right)}\right|/\left(\tau_{\gamma }\zeta^{\left(d\right)}\right)\right)$. Therefore, $\lambda_{k}^{\left(d\right)}$ is updated conditional on all parameters excluding *all* $w_{k,j_{k}}^{\left(d\right)}$, *j*_*k*_ = 1, 2, …, *p*_*k*_. Its distribution can be acquired by noting that $\gamma_{k,j_{k}}^{\left(d\right)}|\tau_{\gamma ,\zeta }(d)$, $\lambda_{k}^{\left(d\right)}\sim DE\left(\mu =0,b=\tau_{\gamma }\zeta^{\left(d\right)}/\lambda_{k}^{\left(d\right)}\right)$ (Park and Casella, 2008), where *DE* stands for double exponential or Laplace distribution.
- for each *k* = 1, 2, …, *K*, *d* = 1, 2, …, *D* and *j*_*k*_ = 1, 2, …, *K*, we use $B_{k,j_{k}}^{\left(d\right)}$ to denote the $j_{k}^{th}$ slice of tensor $B_{k}^{\left(d\right)}$ along mode *k*, which is a (*K* − 1)-mode tensor. Then, $\text{Vec}\left(B_{k,j_{k}}^{\left(d\right)}\right)|\cdot \sim N\left(\mu^{*},\Sigma^{*}\right)$, for $\Sigma^{*}={\left(\Sigma_{\pi }^{-1}+\left(\sum_{i=1}^{N}\Psi_{i}\Psi_{i}^{T}\right)/\tau^{2}\right)}^{-1}$, and $\mu^{*}=\Sigma^{*}\left(\Sigma_{\pi }^{-1}\mu_{\pi }+\left(\sum_{i=1}^{N}\Psi_{i}R_{i,\Psi }\right)/\tau^{2}\right)$, where
  - Σ_*π*_ is a diagonal matrix of dimension $\left(\prod_{k=1}^{K}p_{k}\right)/p_{k}$ with repeated entry $\sigma_{k}^{2}\zeta^{\left(d\right)}$,
  - ***μ***_*π*_ is a constant vector of length $\left(\prod_{k=1}^{K}p_{k}\right)/p_{k}$ with entry $\gamma_{k,j_{k}}^{\left(d\right)}$,
  - $\Psi_{i}=\text{vec}\left(B_{1}^{\left(d\right)}\circ \ldots \circ B_{k-1}^{\left(d\right)}\circ B_{k+1}^{\left(d\right)}\ldots B_{K}^{\left(d\right)}\circ X_{i}\right)$, and
  - $R_{i,\Psi }=Y_{i}-\alpha -C_{i}^{T}\delta -{\langle X_{i},\sum_{r\neq d}B_{1}^{\left(r\right)}\circ B_{2}^{\left(r\right)}\circ \ldots \circ B_{K}^{\left(r\right)}\rangle }_{F}$ is the residual excluding component *d* of the coefficient tensor.
- for each *d* = 1, 2, …, *D*, we update *ζ*^(*d*)^ from its full conditional, and then ensure that ***ζ*** sums to 1, by dividing all its entries with $\sum_{d=1}^{D}\zeta^{\left(d\right)}$. The *ζ*^(*d*)^ update is from $\zeta^{\left(d\right)}|\cdot \sim giG\left(p^{*},a^{*},b^{*}\right)$, where $p^{*}=\alpha /D-K\left(\prod p_{k}+\sum p_{k}\right)/2$, *a** = 0, and $b^{*}=\sum_{k,\underset{˜}{j}}{\left(\beta_{k,\underset{˜}{j}}^{\left(d\right)}-\gamma_{k,j_{k}}^{\left(d\right)}\right)}^{2}/\sigma_{k}^{2}+\sum_{k,j_{k}}\gamma_{k,j_{k}}^{2}/\left(\tau_{\gamma }w_{k,j_{k}}^{\left(d\right)}\right)$.

## Appendix D. Additional Simulation Results

### D.1 Comparing Softer and PARAFAC in Identifying Important Entries of the Tensor Predictor

In Section 5.1 we presented an evaluation of the relative performance of Softer and hard PARAFAC in identifying important entries. There, we showed that Softer has significant lower FPR indicating that the two methods systematically disagree in the entries of the tensor predictor they identify as important. In order to study their disagreement, for each entry of the tensor predictor we calculate the percentage of data sets for which Softer or hard PARAFAC identifies the entry as important while the other does not. We plot the results in Figure D.1 as a function of the entry’s true coefficient. We see that the entries that Softer identifies as important and hard PARAFAC does not happen uniformly over the entries’ true coefficient. In contrast, when hard PARAFAC identifies an entry as important and Softer does not, it is more likely that the coefficient of this entry will be in reality small or zero.

When further investigating this behavior of the hard PARAFAC, we identified that the entries that it identifies as significant in disagreement to Softer are most often the ones that attribute to the coefficient tensor’s block structure. This is evident in Figure D.2 where we see that the entries with high identification by PARAFAC in contrast to Softer are the ones at the boundary of the truly non-zero entries.

### D.2 Simulation Results with Alternative Coefficient Tensors

For a tensor predictor of dimension 32 × 32 we also considered three alternative coefficient matrices. The constant squares represent a rank-3, sparse scenario. Both the hard PARAFAC and the Lasso are expected to perform well in this situation, and we are interested in investigating Softer’s performance when softening is not necessary. The varying feet and dog scenarios are scenarios similarly to the dog and feet of the main text but for non-zero entries varying between 0.5 and 1.

Figure D.3 shows the true and average estimated coefficient matrices in these additional simulations. Even though we plot the true, Softer, and hard PARAFAC matrices using a common scale, we plot the expected coefficients employing Lasso using a different color scale. That is because, we want to show that Lasso gets the correct structure, on average, but largely underestimates coefficients due to the assumption of sparsity. Further, Table D.1 reports the average absolute bias, root mean squared error and 95% coverage of the truly zero, and truly non-zero coefficients, and the prediction mean squared error. When the true underlying hard PARAFAC structure is correct, Softer is able to revert back to its hard version, as is evident by the simulation results for the constant squares coefficient matrix. Further, Softer performs better than the hard PARAFAC for the varying feet and varying dog scenarios. In all three scenarios, Softer has the best out-of-sample predictive ability.

### D.3 Simulation Results for 32 × 32 Tensor Predictor and Sample Size *n* = 200

Simulation results in this section represent a subset of the scenarios in Section 5.1 (dog, feet, diagonal) but for sample size *n* = 200. The general conclusions from Section 5.1 remain even when considering a smaller sample size. Figure D.4 shows a plot similar to the one in Figure 5 including the true coefficient matrices and average posterior mean or penalized estimate across data sets. Again, the color scale of the results is the same for Softer and hard PARAFAC, but is different for the Lasso. Using different scales facilitates illustration of the underlying structure the methods estimate, even though different methods estimate different magnitude of coefficients. For example, Lasso captures the feet structure correctly, but non-zero coefficients are greatly underestimated around 0.1 (instead of 1). In contrast, in the truly sparse, diagonal scenario, Lasso estimates non-zero coefficients at about 0.8 whereas Softer estimates them to be close to 0.3, and hard PARAFAC near 0.06.

**Table D.1:** Average bias, root mean squared error, frequentist coverage of 95% credible intervals among truly zero and truly non-zero coefficient entries, and predictive mean squared error for Softer, hard PARAFAC and Lasso for the simulation scenario with tensor predictor of dimensions 32 × 32 and sample size *n* = 400. Bold text is used for the approach performing best in each scenario and for each metric.

			Softer	PARAFAC	Lasso
constant squares	Truly zero	bias	0.0016	0.0014	**0.0013**
		rMSE	0.016	**0.015**	0.016
		coverage	99.2%	99.7%	-
	Truly non-zero	bias	0.0211	**0.017**	0.073
		rMSE	**0.06**	0.076	0.093
		coverage	**90.9%**	79.6%	-
	Prediction	MSE	**0.79**	1.25	1.32
varying feet	Truly zero	bias	0.06	0.076	**0.014**
		rMSE	**0.123**	0.145	0.169
		coverage	96.4%	90.7%	–
	Truly non-zero	bias	**0.165**	0.205	0.569
		rMSE	**0.244**	0.279	0.661
		coverage	**87.7**%	73.8%	–
	Prediction	MSE	**40.79**	55.19	194
varying dog	Truly zero	bias	0.071	0.091	**0.013**
		rMSE	0.159	0.176	**0.152**
		coverage	98.3%	92.7	–
	Truly non-zero	bias	**0.263**	0.321	0.554
		rMSE	**0.358**	0.398	0.644
		coverage	**81.2**	63.2	–
	Prediction	MSE	**67.6**	85.3	159.2

One of the main conclusions is that Softer deviates less from hard PARAFAC when the (*n*, *p*) ratio is small, which is evident by the more rectangular structure in the mean coefficient matrix for the dog scenario, and the stronger shrinkage of the non-zero coefficients in the diagonal scenario. Further, Table D.2 show the average absolute bias and root mean squared error for estimating the coefficient matrix entries, and the prediction mean squared error.

**Table D.2:** Mean bias and rMSE among truly zero and truly non-zero coefficient entries (presented as bias (rMSE)), and average and IQR of the predictive mean squared error (presented as average (IQR)) for tensor predictor of dimensions 32 × 32 and *n* = 200. Bold text is used for the approach performing best in each scenario.

		Softer	PARAFAC	Lasso
feet	Truly zero	0.06 (0.14)	0.065 (0.15)	**0.01 (0.118)**
	Truly non-zero	**0.216** (0.332)	0.229 **(0.328)**	0.535 (0.585)
	Prediction	95.4 (76.6, 111.1)	**94 (78.8, 107.5)**	312 (299, 323)
dog	Truly zero	0.042 (0.155)	0.087 (0.164)	**0.017 (0.155)**
	Truly non-zero	**0.171** (0.264)	0.174 **(0.254)**	0.248 (0.305)
	Prediction	110.6 (99.6, 121.1)	**101.2 (94, 107.8)**	177.3 (170.9, 183.4)
diagonal	Truly zero	0.005 (0.054)	0.003 (0.038)	**0.002 (0.02)**
	Truly non-zero	0.753 (0.773)	0.945 (0.948)	**0.149 (0.181)**
	Prediction	22.76 (21.04, 24.29)	31.08 (30.1, 31.78)	**2.00 (1.57 2.32)**

### D.4 Simulation Results for Alternative Rank of the Hard PARAFAC

In order to investigate the reliance of hard PARAFAC and Softer on the rank of the PARAFAC approximation, we considered the simulation scenarios of Section 5.1 for *D* = 7. Results are shown in Table D.3. (Simulations comparing the performance of Softer and the hard PARAFAC for varying *D* under an alternative scenario are included in Appendix D.5).

Comparing Table D.3 to the results in Section 5.1 we see that the performance of Softer is remarkably unaltered when *D* = 3 or *D* = 7. Perhaps the only difference is in the dog simulations where bias and mean squared error for the truly zero coefficients is slightly increased when *D* = 7. This indicates that Softer is robust to the specification of the rank of the underlying hard PARAFAC.

In contrast, the hard PARAFAC approach shows improvements in performance when *D* = 7 compared to *D* = 3. This is evident when examining the bias and rMSE for the coefficient entries in the feet, dog, and diagonal scenarios. Specifically, increasing the rank of the hard PARAFAC from *D* = 3 to *D* = 7 leads to reductions of up to 15% in absolute bias and 10% in rMSE. When examining the mean estimated coefficient matrices (not shown) we see the improvements in estimation are in picking up the toes (in the feet scenario) and the eyes (in the dog scenario). However, the improvements in performance are quite minor. We suspect that the reason is that the singular values of the true coefficient matrices decrease slowly after the first three, indicating that adding a few additional components does not drive much of the approximation. Relatedly, it is likely that the Dirichlet prior on ***ζ*** in Guhaniyogi et al. (2017), along with a prior on Dirichlet parameter *α*, reduces the approximation rank to effective values smaller than 7.

**Table D.3:** Simulation results for Softer and hard PARAFAC with *D* = 7.

			Softer	PARAFAC
squares	Truly zero	bias	0.003	0.005
		rMSE	0.034	0.049
		coverage	99.5%	98.6%
	Truly non-zero	bias	0.085	0.104
		rMSE	0.111	0.146
		coverage	79.6%	70.5%
	Prediction	MSE	5.17	8.76
feet	Truly zero	bias	0.035	0.041
		rMSE	0.092	0.104
		coverage	97.3%	96.7%
	Truly non-zero	bias	0.112	0.128
		rMSE	0.181	0.199
		coverage	89.2%	85.2%
	Prediction	MSE	30.69	37.15
dog	Truly zero	bias	0.079	0.078
		rMSE	0.138	0.138
		coverage	92.8%	94.7%
	Truly non-zero	bias	0.084	0.095
		rMSE	0.157	0.166
		coverage	92.4%	90.3%
	Prediction	MSE	32.49	36.68
diagonal	Truly zero	bias	0.002	0.005
		rMSE	0.02	0.06
		coverage	100%	99.9%
	Truly non-zero	bias	0.113	0.852
		rMSE	0.128	0.861
		coverage	93.3%	4.1%
	Prediction	MSE	1.43	28.09

Despite the improvements in performance for the hard PARAFAC when the rank is increased, Softer with either rank (*D* = 3 or 7) outperforms the hard PARAFAC. We suspect that the reason is that Softer allows for deviations from the underlying PARAFAC with *D* = 3 across any singular value (see argument corresponding to Figure 4).

### D.5 Comparing Softer and the Hard PARAFAC when Varying the Underlying Rank *D* and the True Rank of the Coefficient Tensor

Here we provide additional simulation results from Section 5.2 for more choices of the algorithmic rank *D* of the hard PARAFAC and Softer. Figure D.5 shows the results. Of course, as the true rank of the coefficient tensor increases, the performance of both methods deteriorates, irrespective of the algorithmic rank used. That is expected since the problem becomes harder with more parameters required to estimate the coefficient matrix, and sample size equal to 200. In agreement to the results in Section 5.2, the performance of Softer is generally better than that of the hard PARAFAC, especially when the true rank is larger than the value of *D* used.

Next, we compare how the same method performs for different values of *D*. The performance of the hard PARAFAC with higher values of *D* is consistently better than the same method’s performance for lower values of *D*. In contrast, Softer performance is strikingly similar across most values of *D*, illustrated by essentially indistinguishable lines, especially for larger values of the true coefficient tensor. These results again illustrate that the results from Softer are robust to the choice of *D*.

## Appendix E. Additional Information on our Brain Connectomics Study

### E.1 Outcome Information for our Brain Connectomics Study

Table E.1 shows the full list of outcomes we considered in our analysis. Both binary and continuous outcomes are considered. Information includes the category of trait. C/B represents continuous and binary outcomes.

**Table E.1:** List of outcomes with description and descriptive statistics.

Category	Name	Description	#obs	Type	Mean (SD)
Cognition	ReadEng AgeAdj	Age adjusted reading score	1,065	C	107.1 (14.8)
	PicVocab AgeAdj	Age adjusted vocabulary comprehension	1,065	C	109.4 (15.2)
	VSPLOT TC	Spatial Orientation (Variable Short Penn Line Orientation Test)	1,062	C	15 (4.44)
Motor	Endurance AgeAdj	Age Adjusted Endurance	1,063	C	108.1 (13.9)
	Strength AgeAdj	Age Adjusted Strength	1,064	C	103.6 (20.1)
Substance Intake	Alc 12 Drinks Per Day	Drinks per day	1,010	C	2.3 (1.57)
	Alc Hvy Frq 5plus	Frequency of 5+ drinks	1,011	C	3 (1.44)
	TB DSM Difficulty Quitting	Tobacco difficulty quitting	280	B	74.6%
	Mj Ab Dep	Marijuana Dependence	1,064	B	9.3%
Psychiatric and	ASR Intn T	Achenbach Adult Self-Report (Internalizing Symptoms)	1,062	C	48.5 (10.7)
Life function	ASR Oth Raw	Achenbach Adult Self-Report (Other problems)	1,062	C	9.1 (4.6)
	Depressive Ep	Has the participant experienced a diagnosed DSMIV major depressive episode over his/her lifetime	1,035	B	9.2%
Emotion	AngHostil Unadj	NIH Toolbox Anger and Affect Survey (Attitudes of Hostility)	1,064	C	50.5 (8.58)
Personality	NEOFAC Agreeableness	“Big Five” trait: Agreeableness	1,063	C	32 (4.93)
Health	BMI	Body Mass Index	1,064	C	26.4 (5.1)

### E.2 Additional Results

Figure E.1 shows predictive power of (1) symmetric Softer with *D* = 3 and (2) *D* = 6, (3) standard Softer ignoring symmetry with *D* = 6, (4) hard PARAFAC and (5) Lasso. Results from (2), (4), and (5) are also shown in Figure 7. Comparing results from (1) and (2) we see that increasing the rank from *D* = 3 to *D* = 6 improved predictions for a subset of outcomes. Ignoring the symmetry of the tensor predictor performed sometimes better and sometimes worse than accounting for symmetry directly into Softer, showing that the two approaches perform comparably for prediction (comparing (2) and (3)).

The two versions of Softer with *D* = 3 and *D* = 6 returned related results for the identified important entries. Table E.2 shows the connections identified based on Softer for *D* = 3. When comparing the identified entries between symmetric Softer with rank 3 and 6, we see that the important connection for strength in Table 4 is also identified by Softer with *D* = 3, using either count or CSA as the tensor predictor. However, Softer with *D* = 3 identified a larger number of important connections for predicting strength than Softer with *D* = 6, though all of the connections involved the Precuneus region. The 3 connections identified by Softer with *D* = 3 using CSA were among the 5 connections identified by the same method using count as the predictor, an indication of stability in the identified brain connections across different features of brain connections. These results indicate the interplay between increasing the number of parameters to be estimated when increasing the rank, with the increased flexibility that increasing the rank provides to estimation. No important connections were identified for having had a depressive episode when using Softer with *D* = 3, and the identified connection for predicting VSPLOT includes the Banks of Superior Temporal Sulcus as one of the two regions, though in the different hemisphere and in combination with a different region for *D* = 3 and *D* = 6. It is also promising that the 2 out of 3 connections identified by Softer with *D* = 3 for CSA tensor predictor and endurance as the outcome correspond to the same pair of regions, though in the opposite hemisphere.

### E.3 Reproducibility of Identified Connections Across Subsamples

We fit Softer with *D* = 6 on 15 randomly chosen subsamples of our data which included 90% of the observations. Since the sample size is smaller in the subsamples, we expect our power to detect important connections to also decrease. We investigate in how many of the subsamples we identify the connections marked as “important” in the full data set (with the same method), shown in Table 4.

**Table E.2:** Identified connections based on Softer with *D* = 3.

Outcome	Feature	ROI 1	ROI 2	
Strength	Count	(rh) Precuneus	(rh) Superior Parietal	**
			(lh) Superior Frontal	*
			(rh) Caudal Middle Frontal	*
			(rh) Isthmus of cingulate gyrus	
			(rh) Pericalcarine	
	CSA	(rh) Precuneus	(rh) Superior Parietal	**
			(lh) Superior Frontal	*
			(rh) Caudal Middle Frontal	*
VSPLOT	CSA	(rh) Banks of Superior Temporal Sulcus	(lh) Superior Frontal	
Endurance	CSA	(lh) Cuneus	(lh) Pericalcarine	*
		(lh) Cuneus	(rh) Pericalcarine	*
		(lh) Insula	(rh) Inferior Parietal	

**: connections that are related to those identified based on Softer with *D* = 6.

*: connections that are also identified based on alternative feature, outcome, or hemisphere.

#### Strength.

Using the count of streamlines as the tensor predictor, Softer with *D* = 6 identified one important connection between the ROIs Precuneus and Superior Parietal, both in the right hemisphere. The same connection was also identified in 10 out of 15 subsamples, while Softer did not identify any important connection in 4 of the subsamples. These results indicate that the identified connection for strength based on the count of streamlines stated in Table 4 is reproducible across subsamples.

#### Depressive Episode.

Out of 15 subsamples, Softer with *D* = 6 identified important connections in 6 subsamples when using count of streamlines, and in 7 subsamples when using CSA as the tensor predictor. The connection between the Parahippocampal in the left hemisphere and the Lateral Orbitofrontal in the right hemisphere, was identified in all 13 instances with identified connections, based on either predictor (and in agreement with the results in Table 4). What’s more, *any* of the connections identified across *any* subsample and for *either* predictor involved at least one of these two regions, implying that the importance of these two regions in predicting whether an individual has had a depressive episode is reproducible.

#### VSPLOT.

Using CSA as the tensor predictor, Softer with *D* = 6 identified no connections in any of the subsamples. This might imply that the connection in Table 4 is not reproducible, or that the reduced sample size in the subsamples does not allow us to identify it.

## Appendix F. Symmetric and Semi-Symmetric Soft Tensor Regression

### F.1 Softer for Symmetric 2-mode Predictor

We start again from model Equation 2 with $Y_{i}=\mu +C_{i}^{T}\delta +{\langle X_{i},B\rangle }_{F}+\epsilon_{i}$. However, now *X*_*i*_ is an *R* × *R* symmetric matrix with ignorable diagonal elements. This means that we can think of ***B*** as a real symmetric matrix with ignorable diagonal elements.

#### F.1.1 Eigenvalue decomposition of real symmetric matrices

We can still approximate ***B*** in the same way as in PARAFAC (SVD) by writing $B=\sum_{d=1}^{D}\gamma_{1}^{\left(d\right)}⊗\gamma_{2}^{\left(d\right)}$ for some *D* large enough and $\gamma_{1}^{\left(d\right)}$, $\gamma_{2}^{\left(d\right)}\in {\mathbb{R}}^{R}$. However, this would not enforce that ***B*** is symmetric, since $B_{j_{1}j_{2}}=\sum_{d=1}^{D}\gamma_{1,j_{1}}^{\left(d\right)}\gamma_{2,j_{2}}^{\left(d\right)}\neq \sum_{d=1}^{D}\gamma_{1j_{2}}^{\left(d\right)}\gamma_{2j_{1}}^{\left(d\right)}=B_{j_{2}j_{1}}$. This implies that the entries of ***B*** would only be identifiable up to $B_{j_{1}j_{2}}+B_{j_{2}j_{1}}$.

Since ***B*** is a real symmetric matrix, it is diagonalizable and it has an eigenvalue decomposition. Therefore, we can think of approximating ***B*** using

(F.1)
$$B=\overset{D}{\underset{d=1}{\sum }}\xi^{\left(d\right)}\gamma^{\left(d\right)}⊗\gamma^{\left(d\right)},$$

for sufficiently large *D*, where $\gamma^{\left(d\right)}\in {\mathbb{R}}^{R}$ and $\xi^{\left(d\right)}\in \mathbb{R}$. Note that the vectors *γ*^(*d*)^ here resemble the ones in the PARAFAC decomposition, but they are the same across the two tensor modes (matrix rows and columns).

The main difference between using the eigenvalue-based approximation in Equation F.1, compared to the PARAFAC-based approximation is the inclusion of the parameters *ξ*^(*d*)^. Here, *ξ*^(*d*)^ are necessary in order to have a eigenvalue decomposition employing vectors with real entries. In fact, excluding *ξ*^(*d*)^ from Equation F.1 can only be used to approximate positive definite symmetric matrices. To see this, take vector $\upsilon \in {\mathbb{R}}^{R}$. Then,

$$\upsilon^{T}\left(\overset{D}{\underset{d=1}{\sum }}\gamma^{\left(d\right)}⊗\gamma^{\left(d\right)}\right)\upsilon =\upsilon^{T}\left(\overset{D}{\underset{d=1}{\sum }}\gamma^{\left(d\right)}\gamma^{(d)T}\right)\upsilon =\overset{D}{\underset{d=1}{\sum }}\upsilon^{T}\gamma^{\left(d\right)}{\left(\upsilon^{T}\gamma^{\left(d\right)}\right)}^{T}=\overset{D}{\underset{d=1}{\sum }}{\left(\upsilon^{T}\gamma^{\left(d\right)}\right)}^{2}\geq 0$$

#### F.1.2 Soft eigenvalue-based tensor regression

In the case of tensor predictors without symmetries, Softer was built based on the PARAFAC (multimodal equivalent to SVD) approximation of the coefficient tensor. Instead, for symmetric matrices, Softer is based on the eigenvalue decomposition while still allowing for deviations in row (and column)-specific contributions. However, these deviations also have to account for the symmetric nature of the tensor predictor.

Write $B=\sum_{d=1}^{D}\xi^{\left(d\right)}B_{1}^{\left(d\right)}\circ B_{2}^{\left(d\right)}$ similarly to Equation 5, and assume prior distributions on all parameters as in Section 4.3. Note that the parameters *γ*^(*d*)^ are not forced to be of norm 1 (as in classic eigenvalue decomposition), and they can have any magnitude. This allows us to restrict the parameters *ξ*^(*d*)^ to be in {−1, 1}, and base shrinkage of unnecessary ranks on shrinkage of the vectors *γ*^(*d*)^. Therefore, we assume a Bernoulli(0.5) distribution over {−1, 1} for parameters *ξ*^(*d*)^.

However, even though the symmetry of the underlying decomposition is enforced based on *γ*^(*d*)^, we need to ensure that it is also enforced when writing $B=\sum_{d=1}^{D}\xi^{\left(d\right)}B_{1}^{\left(d\right)}\circ B_{2}^{\left(d\right)}$ using $B_{1}^{\left(d\right)}$, $B_{2}^{\left(d\right)}$. Note that the Softer framework assumes that entries $\beta_{1,j_{1}j_{2}}^{\left(d\right)}$ are centered around $\gamma_{j_{1}}^{\left(d\right)}$, and similarly entries $\beta_{2,j_{1}j_{2}}^{\left(d\right)}$ are centered around $\gamma_{j_{1}}^{\left(d\right)}$. However, doing so does not necessarily lead to symmetric matrices ***B*** since

$$B_{j_{1}j_{2}}=\overset{D}{\underset{d=1}{\sum }}\beta_{1,j_{1}j_{2}}^{\left(d\right)}\beta_{2,j_{1}j_{2}}^{\left(d\right)}\neq \overset{D}{\underset{d=1}{\sum }}\beta_{1,j_{2}j_{1}}^{\left(d\right)}\beta_{2,j_{2}j_{1}}^{\left(d\right)}=B_{j_{2}j_{1}}.$$

We enforce symmetry of ***B*** by focusing only on the lower-triangular part. Softer for symmetric matrix predictor specifies row *i*’s contributions to entries ***B***_*ij*_, $\beta_{1,ij}^{\left(d\right)}$, as centered around $\gamma_{i}^{\left(d\right)}$ only for *i* > *j*. Then, for *j* > *i* we set $\beta_{1,ij}^{\left(d\right)}=\beta_{1,ji}^{\left(d\right)}$. Similarly, column *i*’s contributions to entries ***B***_*ij*_, $\beta_{2,ji}^{\left(d\right)}$ are centered around $\gamma_{i}^{\left(d\right)}$ only for *i* < *j*, and for *j* > *i* we set $\beta_{2,ji}^{\left(d\right)}=\beta_{2,ij}^{\left(d\right)}$. An equivalent way to enforce symmetry on ***B*** is to allow all entries in $B_{1}^{\left(d\right)}$ to have the same form as in Softer, and force $B_{2}^{\left(d\right)}={\left(B_{1}^{\left(d\right)}\right)}^{T}$.

#### F.1.3 Note on implementation using RStan

Note that RStan cannot directly handle discrete parameters as *ξ*^(*d*)^. The most common approach to discrete parameters is to specify the likelihood integrating these parameters out. However, this approach is not easily applicable in our setting since *ξ*^(*d*)^ are entangled in the likelihood through their role in the coefficient matrix ***B***. For that reason, we take an alternative approach, and assume that *ξ*^(*d*)^ are continuous and specify a mixture of normals distribution on each of them: *ξ*^(*d*)^ ~ 0.5*N*(−1, 0.001) + 0.5*N*(1, 0.001). Since the parameters *ξ*^(*d*)^ are not directly of interest, and shrinkage of the contributions of component *d* in a rank−*D* decomposition is achieved through the prior on *γ*^(*d*)^, we expect that this approach will closely resemble results from a specification that defines *ξ*^(*d*)^ to be binary taking values in {−1, 1} from a Bernoulli(0.5) distribution.

### F.2 Softer for Semi-Symmetric 3-mode Tensor

In brain connectomics, and specifically in our study of features of brain connections and their relationship to traits, tensor predictors are often of dimensions *R* × *R* × *p* and are semi-symmetric. Semi-symmetry means that the predictor ***X*** is symmetric along its first two modes and $X_{j_{1}j_{2}j_{3}}=X_{j_{2}j_{1}j_{3}}$. An example of such tensor includes *R* brain regions along the first two modes and *p* features of brain connection characteristics along its third mode. When these features are symmetric (feature of connection from region *i* to region *j* is the same as the feature of connection from region *j* to region *i*), the tensor predictor is semi-symmetric. In such cases, the standard Softer approach could be applied, but entries of ***B*** would be identifiable only up to $B_{j_{1}j_{2}j_{3}}=B_{j_{2}j_{1}j_{3}}$. In order to account for the semi-symmetry in ***X*** we can enforce the same type of semi-symmetry in ***B*** by adopting a PARAFAC-eigenvalue decomposition hybrid.

Specifically, assume that ***B*** is a 3-mode semi-symmetric coefficient tensor corresponding to the semi-symmetric predictor ***X***. Then, for sufficiently large *D*, $\gamma^{\left(d\right)}\in {\mathbb{R}}^{R}$ and $\rho^{\left(d\right)}\in {\mathbb{R}}^{p}$, we can write

(F.2)
$$B=\overset{D}{\underset{d=1}{\sum }}\gamma^{\left(d\right)}⊗\gamma^{\left(d\right)}⊗\rho^{\left(d\right)}.$$

This leads to a natural approximation for ***B*** for some value *D* potentially smaller than the true one. Softer for semi-symmetric tensor predictor builds on Equation F.2 while allowing for deviations in the row-specific contributions along the three modes.

We achieve that by specifying $B=\sum_{d=1}^{D}B_{1}^{\left(d\right)}\circ B_{2}^{\left(d\right)}\circ B_{3}^{\left(d\right)}$ for $B_{k}^{\left(d\right)}$ are tensors of dimensions *R* × *R* × *p*. The structure and specification of $B_{k}^{\left(d\right)}$ are as in Section 4.3 with small changes to account for the semi-symmetric structure in ***X*** and ensure that the estimated coefficient tensor is also semi-symmetric. Note that the (*j*_1_, *j*_2_, *j*_3_) entry of ***B*** is equal to

$$B_{j_{1}j_{2}j_{3}}=\overset{D}{\underset{d=1}{\sum }}\beta_{1,j_{1}j_{2}j_{3}}^{\left(d\right)}\beta_{2,j_{1}j_{2}j_{3}}^{\left(d\right)}\beta_{3,j_{1}j_{2}j_{3}}^{\left(d\right)},$$

and we want $B_{j_{1}j_{2}j_{3}}=B_{j_{2}j_{1}j_{3}}$. Borrowing from the symmetric case, we allow all row-specific contributions along mode 1, $\beta_{1,j_{1}j_{2}j_{3}}^{\left(d\right)}$, to vary around the corresponding entry in the decomposition Equation F.2, $\gamma_{j_{1}}^{\left(d\right)}$, and set $B_{2,..j_{3}}=B_{1,..j_{3}}^{T}$. Further, we allow entries $\beta_{3,j_{1}j_{2}j_{3}}^{\left(d\right)}$ to vary around $\rho_{j_{3}}^{\left(d\right)}$ for *j*_1_ < *j*_2_, and set $\beta_{3,j_{1}j_{2}j_{3}}^{\left(d\right)}=\beta_{3,j_{2}j_{1}j_{3}}^{\left(d\right)}$ when *j*_1_ > *j*_2_. Doing so, ensures that $B_{j_{1}j_{2}j_{3}}=B_{j_{2}j_{1}j_{3}}$.
